# MRET: Modified Recursive Elimination Technique for ranking author assessment parameters

**DOI:** 10.1371/journal.pone.0303105

**Published:** 2024-06-13

**Authors:** Ghulam Mustafa, Abid Rauf, Muhammad Tanvir Afzal

**Affiliations:** 1 Department of Computer Science, University of Engineering and Technology, Taxila, Pakistan; 2 Department of computing, Shifa Tameer-e-Millat University, Islamabad, Pakistan; Lodz University of Technology: Politechnika Lodzka, POLAND

## Abstract

In scientific research, assessing the impact and influence of authors is crucial for evaluating their scholarly contributions. Whereas in literature, multitudinous parameters have been developed to quantify the productivity and significance of researchers, including the publication count, citation count, well-known h index and its extensions and variations. However, with a plethora of available assessment metrics, it is vital to identify and prioritize the most effective metrics. To address the complexity of this task, we employ a powerful deep learning technique known as the Multi-Layer Perceptron (MLP) classifier for the classification and the ranking purposes. By leveraging the MLP’s capacity to discern patterns within datasets, we assign importance scores to each parameter using the proposed modified recursive elimination technique. Based on the importance scores, we ranked these parameters. Furthermore, in this study, we put forth a comprehensive statistical analysis of the top-ranked author assessment parameters, encompassing a vast array of 64 distinct metrics. This analysis gives us treasured insights in between these parameters, shedding light on the potential correlations and dependencies that may affect assessment outcomes. In the statistical analysis, we combined these parameters by using seven well-known statistical methods, such as arithmetic means, harmonic means, geometric means etc. After combining the parameters, we sorted the list of each pair of parameters and analyzed the top 10, 50, and 100 records. During this analysis, we counted the occurrence of the award winners. For experimental proposes, data collection was done from the field of Mathematics. This dataset consists of 525 individuals who are yet to receive their awards along with 525 individuals who have been recognized as potential award winners by certain well known and prestigious scientific societies belonging to the fields’ of mathematics in the last three decades. The results of this study revealed that, in ranking of the author assessment parameters, the normalized h index achieved the highest importance score as compared to the remaining sixty-three parameters. Furthermore, the statistical analysis results revealed that the Trigonometric Mean (TM) outperformed the other six statistical models. Moreover, based on the analysis of the parameters, specifically the M Quotient and FG index, it is evident that combining these parameters with any other parameter using various statistical models consistently produces excellent results in terms of the percentage score for returning awardees.

## Introduction

In today’s research landscape, researchers generate an enormous volume of scholarly articles daily [1]. Qualitative evaluation of researchers’ work plays a vital role in addressing crucial aspects of the academic community. These aspects include determining the eligibility for scholarship awards, identifying individuals who have produced influential research studies, selecting editors and reviewers for scientific conferences and journals, and assessing the competence of potential fellows or members of scientific societies [2]. Moreover, such assessments also aid students in choosing suitable supervisors for their research endeavors [3]. To address these issues, the existing techniques employed to evaluate research work vary depending on the specific criteria and merits of the relevant scientific community. There is no universally standardized approach for measuring a researcher’s potential [4]. Numerous quantitative research assessment parameters have been proposed to identify researchers who make innovative and impactful contributions to the scientific community [5]. Each technique proposed in the literature is based on its distinct criteria for determining the significance of a researcher’s work.

Traditionally, researchers’ output has been measured using publication count as a parameter [6]. The total number of publications is considered an indicator of a researcher’s impact. However, the scientific community has raised concerns about relying solely on total publications to determine the impact of researchers [6]. To illustrate this point, Cameron compared the profiles of two different researchers belonging to the database field, the first E.F. Cod and the second Hector Garcia-Molina. The total publications of E.F. Codd are fewer (49) than Garcia, which is 248, but Codd was still considered to be famous or more prominent than Garcia due to winning the Turing award twice (1981, 1994). This scenario highlights the issue of preference for quality over quantity in researcher assessments [7]. To resolve this issue, the scientific community introduced a new factor, the citation count of the researcher’s work. The citation count represents that, number of times the researcher’s work is cited by another researcher [7]. A higher number of citations represents greater recognition in the research community. However, certain limitations exist when we solely depend on citation count; for example, researchers artificially increase their citations via self-citation, the inclusion of negative citations, and the reality of survey papers that receive more citations, which may not accurately reflect the potential of a study.

In response to these challenges, research attention has been focused on introducing indices that consider the quantity and quality factors of research simultaneously. The h index, proposed by Hirsch [8], is one of the most widely used parameters for researcher assessments. The h index evaluates the quality factor of a researcher’s work and has gained global acceptance because of its computational effectiveness. Hirsch emphasized that the h index not only considers current performance but also predicts future impacts. Numerous researchers have discussed and explored the h index, contributing to its widespread adoption. However, some researchers, especially Dienes, have criticized the h index based on their shortcomings. He discussed that additional citations to indexed papers do not contribute to researchers’ impact. Furthermore, in most cases, different authors can have the same h index even with varying numbers of published papers and their respective citations, and vice versa [9].

To address the issues of the h index, more than 70 parameters have been proposed in the literature [10], such as the g-index, k-index, w-index, x-index, and Maxprod. Whenever a new technique is proposed in literature it is typically evaluated on hypothetical or fictional case scenarios. As these techniques are validated across various scenarios or datasets, it is challenging to observe their importance owing to their dependence on specific datasets. Recognizing this challenge, the scientific community has devoted effort to developing efficient methods for rapidly ranking researchers [11]. In recent studies, researchers have conducted empirical evaluations of the h index and its variants to assess their contributions to determining the achievements of award winners in the fields of Mathematics and Neuroscience [5, 12]. These evaluations aimed to gain insights into the effectiveness of these indices, specifically within these fields. By analyzing the performance of these indices in real-world scenarios, researchers have sought to enhance the understanding of their applicability and provide valuable insights for research evaluation in these disciplines.

After conducting a critical analysis of the literature, we identified the following gaps:

- To the best of our knowledge, no studies have ranked a large number of parameters using deep learning and machine learning techniques.
- No studies have combined these parameters using various statistical methods and observed the resulting outcomes.

This study aimed to address these issues. To address the raking problem, we used well-known deep learning multilayer perceptron classifiers. With this classifier, to determine the importance of each feature, we require a feature selection technique. For this, we employ a modified recursive elimination technique in machine learning to extract the importance score for each feature, which constitutes another contribution to our work. By applying this modified recursive elimination technique, we obtained important scores for each parameter. These scores allowed us to rank the parameters based on their relative importance, which is a valuable contribution to the field of author assessment. Furthermore, we address the second point by analyzing the results of the top-ranked parameters using statistical methods. In this task, we combined the top-ranked individual parameters and performed a comprehensive statistical analysis to derive meaningful insights. The integration of statistical methods with ranked parameters constitutes a unique and valuable contribution to the field.

For evaluation purposes, we collected a dataset from the mathematics domain consisting of data from 1050 authors. The dataset comprises 525 non-awardees of data collected from the dataset provided by [12]. To balance the dataset, we added the data of the remaining 525 awardees from the four prominent mathematics societies (AMS, IMU, LMS, and NASL). The awardees data belong to the last three decades. This study addressed the following two questions:

- **RQ1**. Which index has a strong relationship with award winners in the mathematics domain as compared to others?
- **RQ2.** Which statistical methods contribute the most by retrieving the highest number of awardees compared to others?

To address the research question outlined above, this study presents two primary contributions. Firstly, we introduce the MERT technique, aimed at ranking parameters and identifying the most significant index with a strong correlation to award winners. As a result of this endeavor, we have derived the normalized h-index, which emerges as the most robust parameter associated with award recipients compared to others. Secondly, we conduct a thorough statistical analysis to integrate the top-ranked parameters and determine the statistical method that offers the most substantial contribution. This analysis reveals that the trigonometric mean surpasses the performance of the other six statistical models.

The remainder of this paper is organized as follows. First, we provide a brief review of the ranking parameters in the “Literature Review” section. Next, the “Methodology” section presents our proposed approach to ranking the indices and performing statistical analysis. The study results are discussed in the “Results and Discussion” sections. Finally, the “Conclusion” section conclusion section concludes the paper.

## Literature

In the vast realm of scientific research, the need for a universal criterion to evaluate and rank researchers’ scientific performance fairly cannot be overstated. Various parameters come into play when assessing and ranking the scientific performance of a researcher, including publication count, citation count, h index, and its variants. Subjective evaluations conducted by the scientific community are often employed to nominate scholars for academic and professional awards and promotions [13–17]. However, these traditional strategies rely heavily on quantitative metrics such as publication and citation counts, which have been extensively criticized due to their limitations.

A high publication count does not reflect the quality of work; the author may publish their article in a low-impact factor journal or local conferences [18]. Similar to the previous scenario, citation counts individually do not represent research influence, as they can be easily manipulated. The author may cite their articles or articles cited from negative perspectives such practices, increases the number of citations but this does not accurately reflect the impact of their work [19].

To address the shortcomings of conventional measures, Hirsch [8] introduced the h index, which has gained popularity among researchers because of its simplicity. However, Dienes [9]criticized the h index for its shortcomings. One of the drawbacks discussed by Dienes is that increasing the citation of index papers is not reflected in the authors’ impact [20]. Moreover, the h index may not be suitable for evaluating the performance of young researchers who have recently entered the field, as it takes time for their publications to accumulate citations and their h index to rise. Additionally, the h index may inadvertently favor researchers who are not active [21, 22]. As a result, more than 70 alternative parameters parameters have been proposed by researchers to mitigate the shortcomings of the h index, such as the g index, k index, t index, f index so on.

A study conducted by Ayaz and Afzal [23] focused on evaluating the effectiveness of the complete h index, g-index, and h index. By analyzing awardees from mathematical scientific societies, they reported in their result that the complete-h index outperformed the g-index and h index. A similar study conducted by Ayaz et al. [3] examined the h index and its variants in the context of elevating award winners to the top of the rankings and concluded that the h index outperformed other alternatives. Moreover, Ameer et al. in 2019 [5] evaluated quantitative parameters for the field of neuroscience societies and reported that the hg-index and R-index effectively elevated awardees to top positions among researchers. Similarly, Ain et al. in 2019 [12] evaluated scientific quantitative parameters for researchers in mathematics. They established a correlation between the selected parameters and ranked these parameters based on award-winning researchers. However, it is important to note that these studies attempted to establish an association between the h index or its variants and award-winning researchers who were recognized before the introduction of these parameters. Hence, it could be coincidental that they found correlations between awardees and quantitative parameters. To overcome this limitation, Usman et al., 2021 [4] proposed a technique for evaluating the h index and its variants using data from the civil engineering domain. In this approach they have not selected researchers randomly, they specifically chose research data (awardees and non-awardees) within the same period, focusing on researchers who received awards in the civil engineering domain, particularly after 2005. However, their dataset is not yet sufficiently comprehensive to definitively determine which parameters are crucial for award winners. Furthermore, Abdulrahman A. Alshdadi et al. in 2023 [24] proposed rules using deep learning, which are considered as minimum thresholds for qualifying subjective evaluations. This technique uses a different domain dataset for evaluation. Furthermore, Mustafa et al., 2023 [2]. evaluated publication and citation count-based category parameters using a mathematics domain dataset. They reported in their result that the normalized h index outperformed all the other indices belonging to this category.

The literature extensively covers a large number of parameters used to determine and evaluate the value of publications and classify exceptional scholars. Over the past decade, scientists have relied primarily on publications and citations to assess researchers. However, as the field evolved, variants of the h index were introduced without necessarily considering the limitations or background of the study. Often, these methodologies have been developed unconventionally or by using different datasets, making it challenging to discern the individual significance of these techniques. Furthermore, to the best of our knowledge, there are no existing studies in the literature that have ranked such a large number of parameters using deep learning and machine learning techniques. Additionally, we identified a lack of studies that performed statistical analyses by combining these parameters. Therefore, this study aims to address these issues.

## Materials and methods

**Fig 1:**
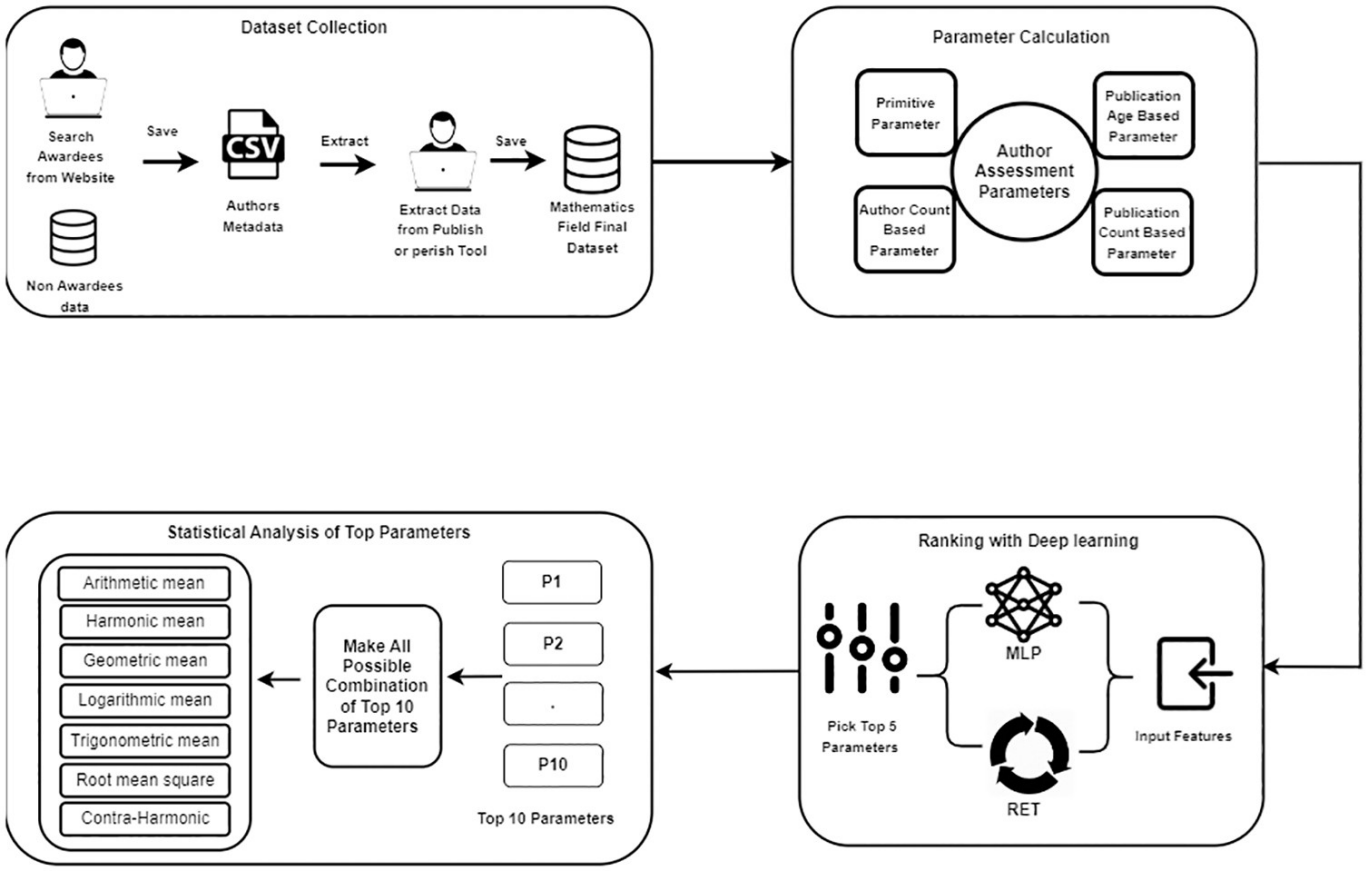
Proposed methodology diagram. This figure illustrates the proposed methodology of our study, which comprises multiple phases. Initially, we collected the dataset from the mathematics domain. Subsequently, we calculated sixty-four parameters. Furthermore, we ranked these sixty-four parameters using a Deep Learning technique, specifically by introducing the MERT technique in this study. Towards the conclusion of the study, we conducted statistical analyses of the top parameter combinations.

The scientific community has proposed several research evaluation metrics to rank researchers. In this study, we aimed to assess and rank these metrics using a deep learning classifier with a modified recursive elimination method. The proposed methodology is illustrated in Fig 1.

### Field selection

To implement the proposed methodology, we must acquire a comprehensive dataset for a particular scientific field. For this purpose, we selected the field of mathematics. This field was selected because it has a long-standing history and has witnessed significant research contributions, making it the best choice for evaluating the proposed methodology. Moreover, we have also noticed that numerous scientific societies within this field grant yearly awards to renowned researchers based on the impact of their work. Furthermore, researchers in this field have not been comprehensively utilized for ranking and evaluating h index variations. Thus, ranking these parameters can be helpful for the scientific community in this field to identify potential researchers and support the development and growth of this domain.

### Dataset collection

**Fig 2:**
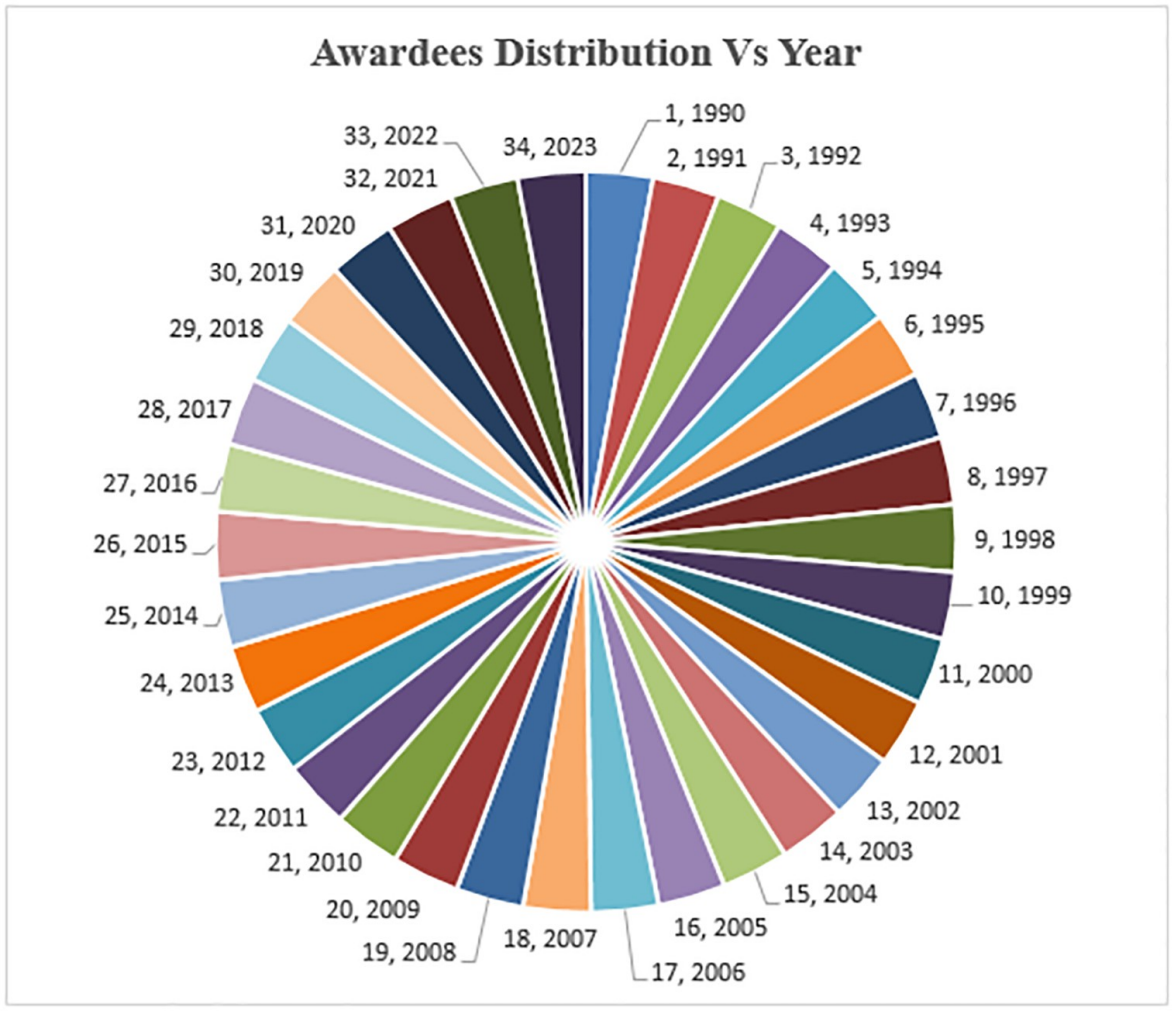
Awardees distribution.

For experimental purposes, we have collected a comprehensive and diverse dataset to evaluate the proposed methodology. This dataset comprises 1050 records, encompassing information from both awardees and non-awardees (https://github.com/ghulammustafacomsat/Mathematics_dataset). Specifically, we have included data from 525 non-awardees and 525 awardees. In this dataset, non-awardees’ data was derived from the dataset utilized by Ain et al., 2019 [12] & Ghani et al., 2019 [25]. However, since the original datasets used by Ain et al., 2019 [12] and Ghani et al., 2019 [25] only contained a limited number of awardee entries, extending until 2013, we expanded our dataset by collecting updated information on awardees until 2023. To accomplish this, we visited various society websites of the mathematics domain and gathered the names and corresponding years of recognition for researchers over the past three decades. The distribution of awards across different years is presented in Fig 2.

To extract awardees data, we utilized the Publish or Perish tool, employing a hold-on strategy that allowed us to collect records of researcher’s even prior to their award-receiving year. Moreover, this tool utilizes a sophisticated algorithm that extracts author metadata from Google Scholar (GS). The Google Scholar platform was selected to extract the data for several reasons. First, it offers broad coverage of academic publications across multiple disciplines. Second, it is accessible to researchers across the world and can retrieve both open-access and paid publications. Moreover, numerous studies have compared Google Scholar with Web of Science, reporting that the average growth rate is 13% higher for the former. Furthermore, Google Scholar’s citations showed an average monthly increase of 1.5% over the past year. Google Scholar is a dynamic platform that receives regular updates, ensuring that the information it provides is up-to-date and relevant Martin et al. 2018 [26]. To balance the dataset, we collected non-awardees in the same proportion as the number of awardees for each specific year. For example, if there were 19 awardees in 2008, we collected data from 19 non-awardees prior to 2008, using the same techniques. The statistics pertaining to the datasets are listed in Table 1.

**Table 1:** Dataset statistics before preprocessing.

Researchers Metadata	Count
No of Authors	1050
No of Awardees	525
No of Non Awardees	525
No of total Citation	14,370,007
No of total Publication	204,896

### Data preprocessing

Before conducting any analysis or evaluation, it is crucial to thoroughly clean the data collected from sources such as Google Scholar. This process aims to eliminate irrelevant or incorrect information, referred to as noise, which can compromise the validity of the results. Moreover, the data cleansing process involves various steps including verifying the correctness of the data and removing duplicate entries. In our extensive research dataset, two critical processes were undertaken to enhance the quality and relevance of the data. First, a filter was applied to ensure that every publication belonged to a mathematical field, eliminating irrelevant or non-mathematical content. This step helped to refine the dataset to focus specifically on the relevant domain. For this task, we have executed several steps such as, 1) Eliminating results containing invalid characters in their titles, 2) Validating results to confirm if the published papers originated from Mathematics journals or conferences, 3) Assessing sample results with domain experts to ensure relevance to the Mathematics domain, and 4) Having domain experts verify the remaining dataset by reviewing the titles of the papers. Subsequently, an author disambiguation process was conducted to identify and eliminate duplicate entries caused by authors publishing under different names. After completing these steps and verifying the aforementioned processes, the characteristics and properties of the final dataset were recorded for evaluation. The resulting dataset and corresponding evaluation results are listed in Table 2.

**Table 2:** Dataset statistics after preprocessing.

Researchers Metadata	Count
No of Authors	638,099
No of Awardees	525
No of Non Awardees	525
No of total Citation	14,369,007
No of total Publication	204,796

### Benchmark data set

**Fig 3:**
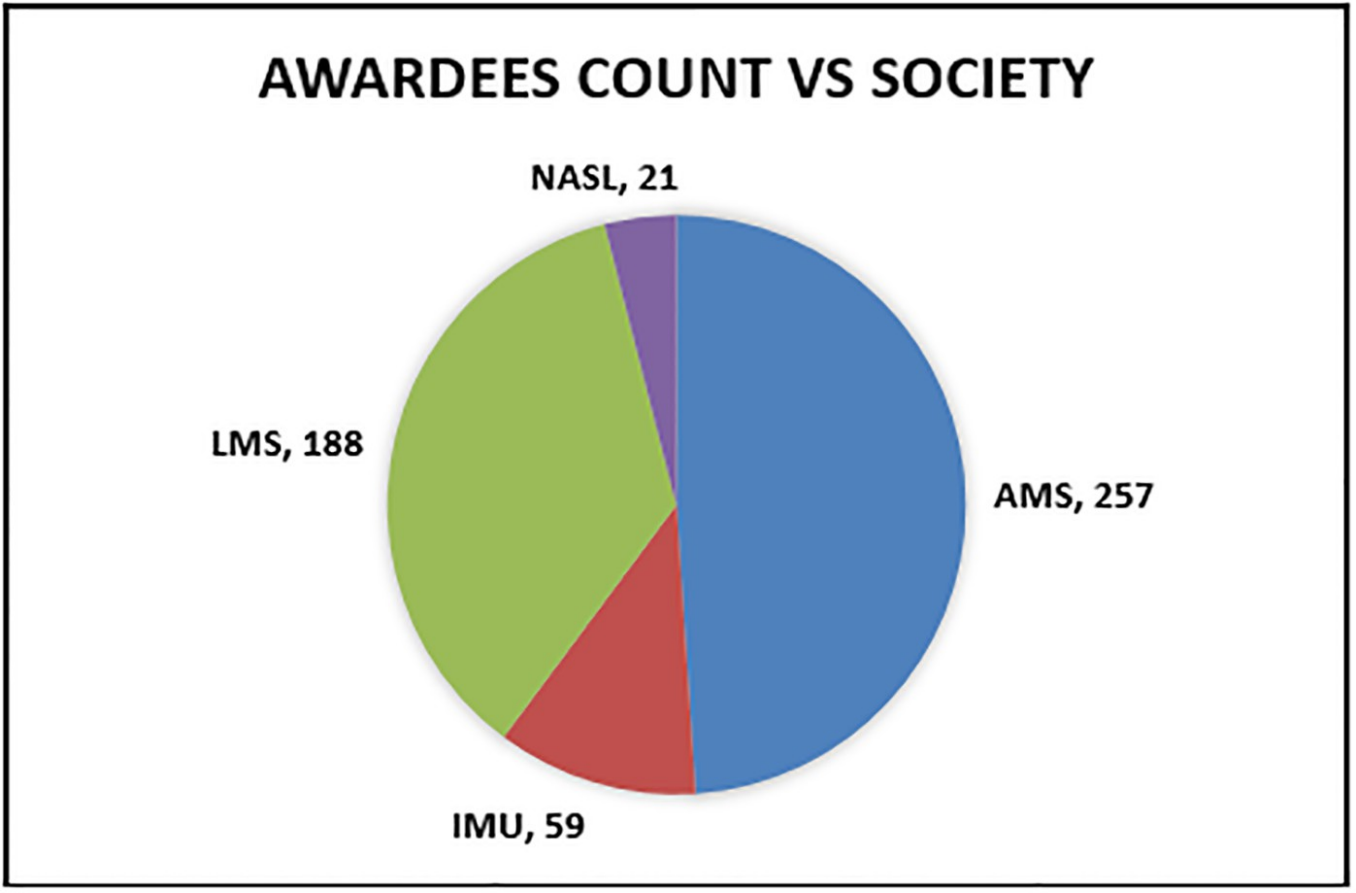
Awardees count against societies.

To conduct a comprehensive evaluation of the various ranking metrics used in our study, we extracted lists of awards from several prestigious mathematical societies. Specifically, we compiled a list of 30 internationally recognized awards that hold significant importance within the mathematical community. These awards are considered notable achievements by mathematicians and researchers. The awards are granted by renowned mathematical societies such as the LMS, IMU, NASL, and AMS. These societies are dedicated to the promotion and advancement of mathematics and support the research and academic pursuits of mathematicians worldwide. Researchers, including Ain et al., 2019 [12], Ayaz and Afzal in 2016 [22], Ghani et al., 2019 [25], and Mustafa et al., 2023 [2] considered awards from these societies as a benchmark for evaluation. The main reason for researchers to consider these societies and their awardees is the lack of alternative benchmarks for evaluating such indices in the field. Fig 3 illustrates the total awardees associated with each society.

### Calculation of indices

Now is this section, research study focused on calculating Sixty-three indices using the collected data. These indices are classified into multiple categories by Bihari et al., 2021 [10]. The list of the categories with respective indices are presented in below (Calculation of the indices are provided in Table 7 in S1 Appendix).

- **Primitive Parameters**
  The parameter belong to primitive parameters are Total Publication, Total Citation, Total years, Cites/Year, Cites/Paper, Author/Paper, Cites/author and Papers/author.
- **Publication and Citation count based parameters**
  The parameter belong to this category are h index, G index, E index, H core citation, A index, R index, P index, M index, F index, T index, Q2 index, Tappered h index, Maxprod, Wu index, Pi index, Weighted h index, H(2) index, Wogienger index, Gh index, Rm index, X index, Hg index, H2 upper index, H2 center index, H2 lower index, K dash index, Rational h index, Real h index, I10 index, Normalized h index, K index, W index and H dash index.
- **Author based Parameters**
  The parameter belongs to author based parameter are HI index, HI norm, Hm index, Gm index, Hf index, Gf index, GF index, K norm index, W norm index, Pure h index, Fractional g index, Fractional h index and Normalized hi index.
- **Age based Parameters**
  The parameter belongs to age based parameters are Platinum h index, M quotient index, AW index, AR index, V index, Ha index, Hc index (Contemporary h index) and AWCR (Age-weighted citation rate).

### Modified Recursive Elimination Techniques (MRET) with MLP

**Fig 4:**
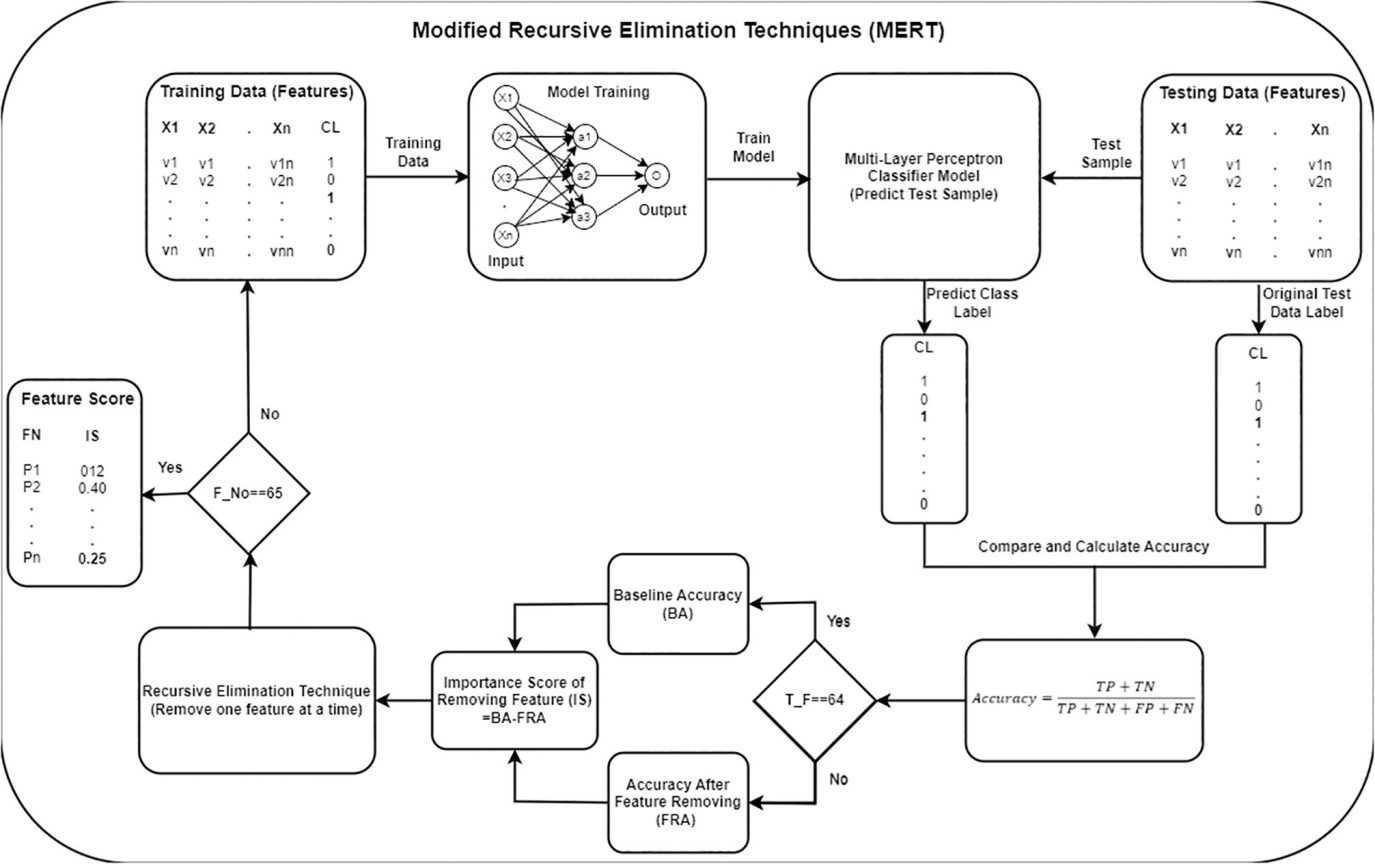
Proposed MERT technique. In MERT, the dataset was initially divided into three samples such training, validation and testing with the ratio of 64:16:20. A MLC was trained, and at last test sample was provided to the trained model. The accuracy achieved during this prediction stage was considered the Baseline Accuracy (BA) when Total feature (T_F) is equal to sixty four. In the next iteration, the feature removal process began. One parameter was removed from the feature list, and the model was trained on the updated feature set. The new accuracy (FRA) obtained from test sample prediction was then subtracted from the BA, give Importance Score (IS) against removed feature. The entire procedure was iterated for each parameter in the dataset. Upon iterating through all features, when the Feature Number (F_No) reached sixty-five, it signified the completion of iterating through all features. The algorithm generated two lists. The first list contains the names of the features, while the second list contains their corresponding importance scores. Based on these importance scores, the parameters were sorted, providing us with a parameter ranking.

In machine learning, the feature-ranking task is crucial because it helps to identify the most impactful feature for different tasks, such as prediction [27], model interpretability [28], and dimensionality reduction [29]. To address the first research question, we propose a technique called the Modified Recursive Elimination Technique (MERT). This technique is a modified version of the well-known feature selection technique used in ML called the Recursive Elimination Technique (RET). This technique is mostly used to identify relevant features that contribute significantly to model’s performance [30]. Furthermore, this technique reduces the dimensionality of the dataset and improves the model interpretability, efficiency, and generalization ability [31]. RET iteratively removes irrelevant or redundant features and focuses on a subset of features that have the most significant impact on the performance of the model. Fig 4 represents the methodology of our Modified Recursive Elimination Techniques (MRET).

In the proposed algorithm, the first step involves dividing the dataset into three samples training, validation and testing with the ratio of 64:16:20. The training dataset was used to train a multilayer perceptron classifier (detailed in the next section) for classification purposes. Subsequently, a validation sample was provided to the trained model during training, and the model predicted the class label for each sample. Furthermore, the test data used for calculating the accuracy of model prediction on unseen data. This accuracy considered as baseline accuracy when all the features were included. The next phase of the algorithm focuses on feature removal. One parameter was removed from the feature list, and the dataset was again divided into training, validation and testing samples by following the same methodology discussed above. The multilayer perceptron classifier is then trained using the updated feature set. Following training, a test sample was utilized to predict the class label, and the accuracy was noted. The new accuracy obtained was subtracted from the baseline accuracy, yielding a subtraction result that served as the importance score for the removed feature. This process was repeated for each parameter with at least five different epoch phases, yielding an importance score. Eq 1 represents the importance score calculation.

$$\begin{array}{c} ImportanceScore=\frac{1}{5}\sum_{i+=20}^{100}\left(BLA_{i}-WOPA_{i}\right) \end{array}$$ (1)

Where i represents the number of epochs, *BLA*_*i*_ represents the baseline accuracy against the *i*^*th*^ phase, and *WOPA*_*i*_ represents the without-parameter accuracy of the *i*^*th*^ phase. The entire process was repeated for each parameter in the dataset. After iterating through all the features, the algorithm generates two lists. The first list contains the names of the features, whereas the second list contains the corresponding importance scores. The Algorithm 1 present the algorithm of the proposed approach.

**Algorithm 1:** Modified Recursive Elimination Techniques (MERT) with MLP

**Input:** Dataset (Features with Class label);     // Features and its Class Label

**Output:** Parameter list with Importance Score against each Parameter

**while** *i* ≤ *len(Features)* **do**

 **if** *i* == *0* **then**

  *X* ← All Parameters Data;     // Load parameters data except class label

  *y* ← Class Label     // Load class label of all records

  *Xtrain*, *Xtest*, *ytrain*, *ytest* ← Split(X, y, 0.20);     // Split the data

  mlp ← BuildMLPClassifier();     // Build Multilayer Perceptron

  mlp.fit(Xtrain, ytrain);     // Model fit on data

  *ypred* ← mlp.predict(Xtest);     // Class Label prediction

  baseAccuracy ← accuracyScore(ytest, ypred);     // Accuracy with all parameters

 **else**

  *X* ← *X*;     // Assign data after removal of parameter

  *y* ← Class Label;     // Load class label of all records

  *Xtrain*, *Xtest*, *ytrain*, *ytest*← Split(X, y, 0.20);     // Split the data

  mlp ← BuildMLPClassifier();     // Build Multilayer Perceptron

  mlp.fit(Xtrain, ytrain);     // Model fit on data

  accuracy ← accuracyScore(ytest, ypred);     // Predict Accuracy after removal of parameter

  parameterName.append(X[i].name);     // Append the Name of Parameters Which can Eliminate from Parameter List

  importanceScore.append(baseAccuracy-accuracy);     // Importance Score of Parameters Which are Eliminating in iteration List

 **end**

  *X* ← All Parameters Data;     // Load all data before removing index parameter in each iteration

  X.remove(i);     // Removing Index parameter

**end**

**return** parameterNameList;     // Return Parameter Name List

**return** ImportanceScoreList;     // Return Importance Score List

### Multilayer perceptron classifier (MLP)

**Fig 5:**
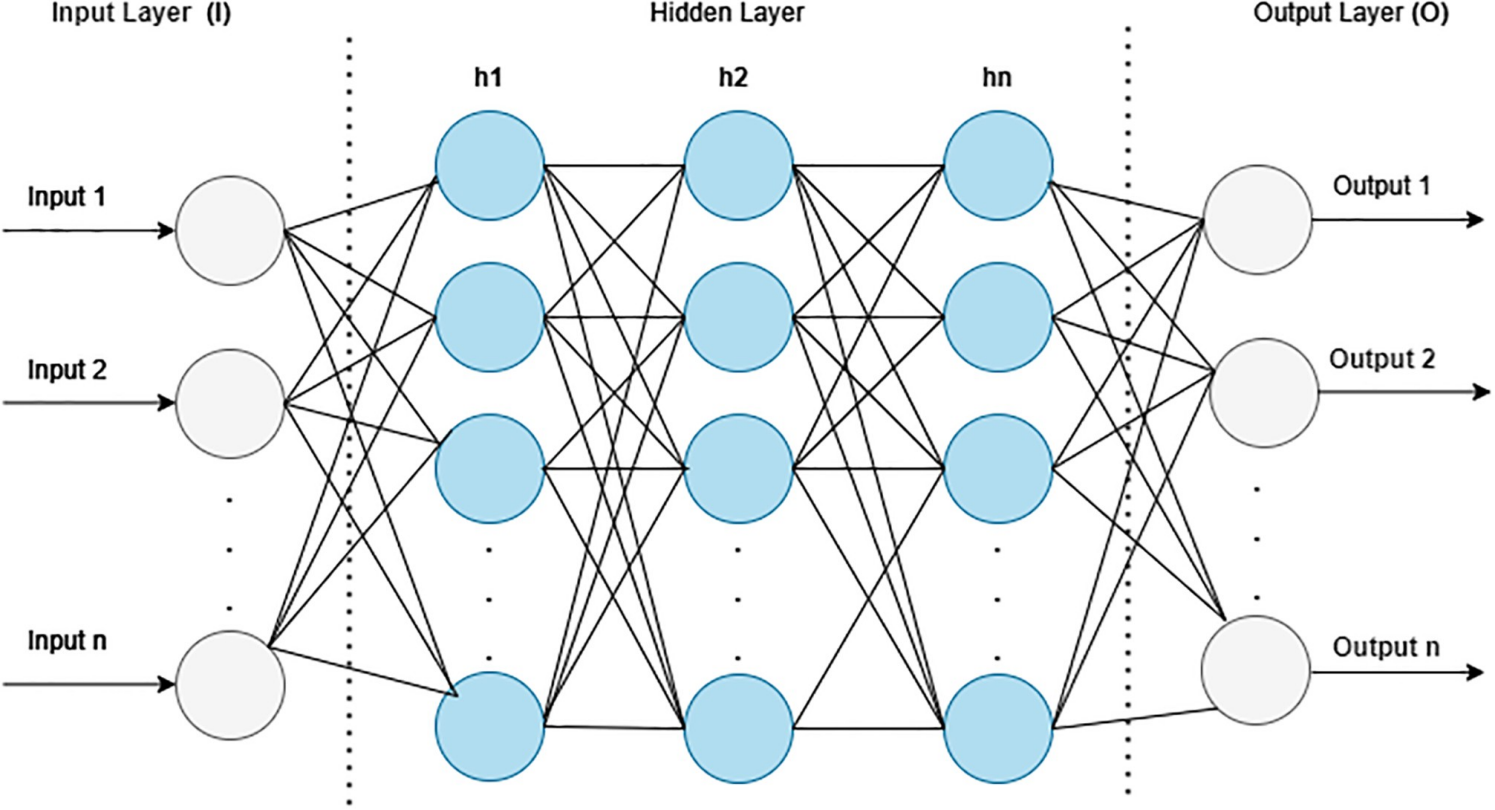
Basic MLP model.

The MLP classifier acted as the backbone of the proposed technique. The MLP is a feed-forward artificial neural network that comprises multiple hidden layers [32] (See Fig 5).

In the classification problem, the total number of features is considered as the number of neurons in an input layer, whereas the total number of classes in which the data are classified is considered as the number of neurons in the output layer. The other layers lie between the input layer and the output layer, which is considered to be a fully connected layer trained using a back propagation algorithm. In the forward propagation phase, the network calculates the output against each layer using an activation function based on the output of the previous layer along with the corresponding weight and bias values, as indicated in Eq 2.

$$\begin{array}{c} Z=WA+b \end{array}$$ (2)

Where Z denotes the output matrix, W denotes the weight matrix, and b denotes the bias vector.

To ensure that the output of the MLP falls within a specific range, an activation function was applied. This function normalizes the output of each layer. By utilizing the activation function, the output of a layer can be transformed into the desired range, as shown in Eq 3.

$$\begin{array}{c} A=g(Z) \end{array}$$ (3)

Where A denotes the activated output matrix.

In our proposed method, for hidden layer we employed a Rectified Linear Unit (ReLU) as the activation while for final output layer we used a Softmax as the activation function. The ReLU activation function defined in Eq 4, transforms values less than zero to zero, while leaving positive values unchanged. This activation function effectively introduces nonlinearity into the network. On the other hand, Softmax, defined by Eq 5, is commonly used in multi-classification tasks. It addresses the limitations of the sigmoid function and ensures that the probabilities of the output layer sum to one. By applying softmax, we can determine the most probable prediction for the given inputs.

$$\begin{array}{c} a_{relu}=max\left(0,z\right) \end{array}$$ (4)

$$\begin{array}{c} a_{soft}=\frac{a^{z_{i}}}{\sum_{j=1}^{j}e^{z_{j}}} \end{array}$$ (5)

Where J denotes class number while *z*^*i*^ denotes *i*^*th*^ output value. Eq 6 represents the loss function, which is used to quantify the error between the two values (predicted and actual values) in the MLP. It serves as a measure of the performance of the model. By calculating the loss, the discrepancy between the predicted and target values can be assessed. Subsequently, a backpropagation algorithm was employed to adjust the weights (w) and biases (b) in the network. This iterative process helps to optimize the model’s performance by minimizing the loss and fine-tuning the parameters of the network.

$$\begin{array}{c} L\left(y,\hat{y}\right)=\frac{1}{m}\sum_{i=1}^{m}{\left(y_{i}-\hat{y_{i}}\right)}^{2} \end{array}$$ (6)

Where m denotes number of samples, y denotes predicted value and $\hat{y}$ denotes actual value. Moreover, in deep-learning models, it is crucial to prevent overfitting. When a deep neural network has excessively deep layers, it can lead to issues such as gradient vanishing or explosion, which adversely affects the model’s performance and contributes to overfitting. To address these problems, a method called batch normalization was introduced by Loffe and Szegedy in 2015 [33]. The goal of batch normalization, as described in Eq 7 counteracted gradient explosions or vanishing. This is achieved by normalizing the output values after each hidden layer, ensuring that they do not become too large or small. The process involves taking the difference between each output and the vector’s mean value, and then dividing it by a standard deviation. In our MLP model, batch normalization was employed after each hidden layer to prevent overfitting effectively.

$$\begin{array}{c} X_{i}=\frac{X_{i}-Mean_{i}}{StandardDeviation_{i}} \end{array}$$ (7)

Where *X*^*i*^ denotes *i*^*th*^ hidden layer’s output matrix, *Mean*_*i*_ is the mean value of *X*^*i*^, and *StandardDeviation*_*i*_ is the standard deviation of *X*^*i*^. In this study, we employed a multilayer perceptron (MLP) as a classifier with 10 hidden layers. The Rectified Linear Unit (ReLU) activation function was utilized in each hidden layer, which consisted of 10 neurons. The selection of the sizes of the hidden layer and neurons was based on multiple experiments. To regularize the network, batch normalization was applied after each hidden layer. The selected features and preprocessed data were input into the neural network through the input layer. The model was trained using forward and backward propagation techniques, whereas the output layer employed the Softmax activation function to generate class probabilities. During the prediction phase, a class probability vector is produced, and the argmax function (See Eq 8) was used to identify the highest probability value and return its corresponding index.

$$\begin{array}{c} Result=max(PredictedVectorSpace) \end{array}$$ (8)

To train our model, we employed the Adam optimization algorithm, which dynamically adjusts the learning rate based on recent weight gradients. Specifically, we used a learning rate of 0.0003, batch size of 64, and conducted training for 100 epochs. Moreover, in this study we divided the dataset into three samples such as Training, Validation, and Testing with the ratio of 64:16:20. In this division, 64% was allocated for actual model training, while 16% was reserved for validation during the training phase. We recognized the importance of having a dedicated testing set separate from the validation set to ensure rigorous evaluation and prevent overfitting. Therefore, in the final stages of our experimentation, we conducted a comprehensive evaluation of the model using the 20% completely unseen data from the testing set. Furthermore, to mitigate overfitting, we implemented the early stopping technique, which halts the training process when signs of overfitting become apparent and restores the best model parameters. The early stopping parameter was set to 40, meaning that if the loss of the validation set did not decrease for more than 40 consecutive epochs, it was determined that the model had overfit. At that point, the training was stopped, and any changes made during the epochs were reversed.

### Ranking of parameter

After obtaining the importance score of each parameter using the MERT algorithm, the parameters were sorted based on their respective scores. This sorting process provides us with parameter ranking.

### Statistical analysis

Statistical methods play a fundamental role in the analysis of data in various research domains [34]. In this study, we employed a range of statistical techniques to gain deeper insights into our research question and derive meaningful conclusions. By harnessing the power of statistical analysis, we were able to systematically examine the data, identify patterns, quantify relationships, and make informed inferences. In this study, we aimed to combine top-ranking parameters using various statistical analysis methods. These methods include the arithmetic mean, contra-harmonic mean, geometric mean, harmonic mean, Lehmer mean, logarithmic mean, root mean square (RMS), and trigonometric mean. By employing these methods, we can obtain a comprehensive understanding of author rankings and assess their significance within the dataset. The calculations for these methods are presented in Table 3. In this study, we employed these sets of statistical methods (presented in Table 3) to analyze the top-ranked parameters in pair form. By utilizing this list of statistical methods, we calculated the corresponding statistical method values for each pair. Subsequently, eight distinct lists were generated for each pair corresponding to each statistical method. Moreover, we compared these lists to discern the most influential statistical method for each pair. Additionally, this analysis allowed us to identify the potential pairings of parameters that exhibit noteworthy patterns or relationships. The results of this study provide valuable insights into the selection of statistical methods and combinations of parameters for further analysis and investigation.

**Table 3:** Statistical methods.

Method Names	Formulas
Arithmetic mean	$Arithmetic=\frac{X_{1}+X_{2}+....+X_{n}}{n}$
Harmonic mean	$HarmonicMean=\frac{n}{\sum_{i=1}^{n}\frac{1}{X_{i}}}$
Contra-harmonic mean	$Contra-HarmonicMean=\frac{\left(X_{1}^{2}\right)+\left(X_{2}^{2}\right)+...+\left(X_{n}^{2}\right)}{\left(X_{1}+X_{2}+X_{3}...+X_{n}\right)}$
Geometric mean	$GeometricMean={\left(X_{1}*X_{2}*X_{3}*...*X_{n}\right)}^{\frac{1}{n}}$
Logarithmic mean	$LogarithmicMean=\left(\frac{1}{n}\right)*\left(\sum_{i=1}^{n}log\left(X_{i}\right)\right))$
Root mean square	$RootMeanSquare=\sqrt{\frac{x_{1}^{2}+x_{2}^{2}+...+x_{n}^{2}}{n}}$ square=
Trigonometric mean	$TrigonometricMean=\frac{\prod_{i=1}^{n}sin\left(x_{i}\right)}{\prod_{i=1}^{n}x_{i}}$

## Results and discussion

The following section outlines the findings obtained in response to the research questions.

### Ranking of parameters

**Fig 6:**
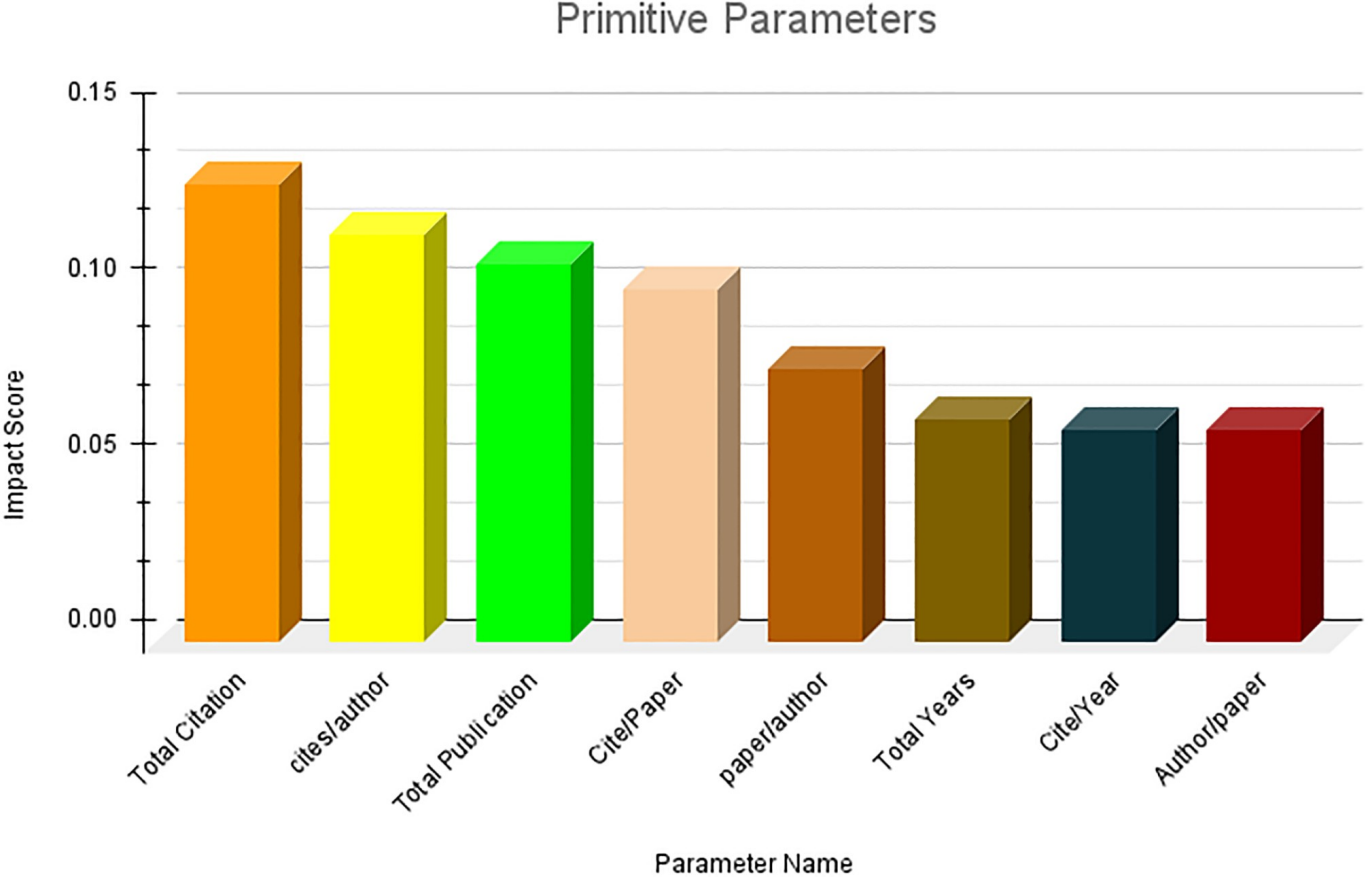
Primitive parameters ranking.

**Fig 7:**
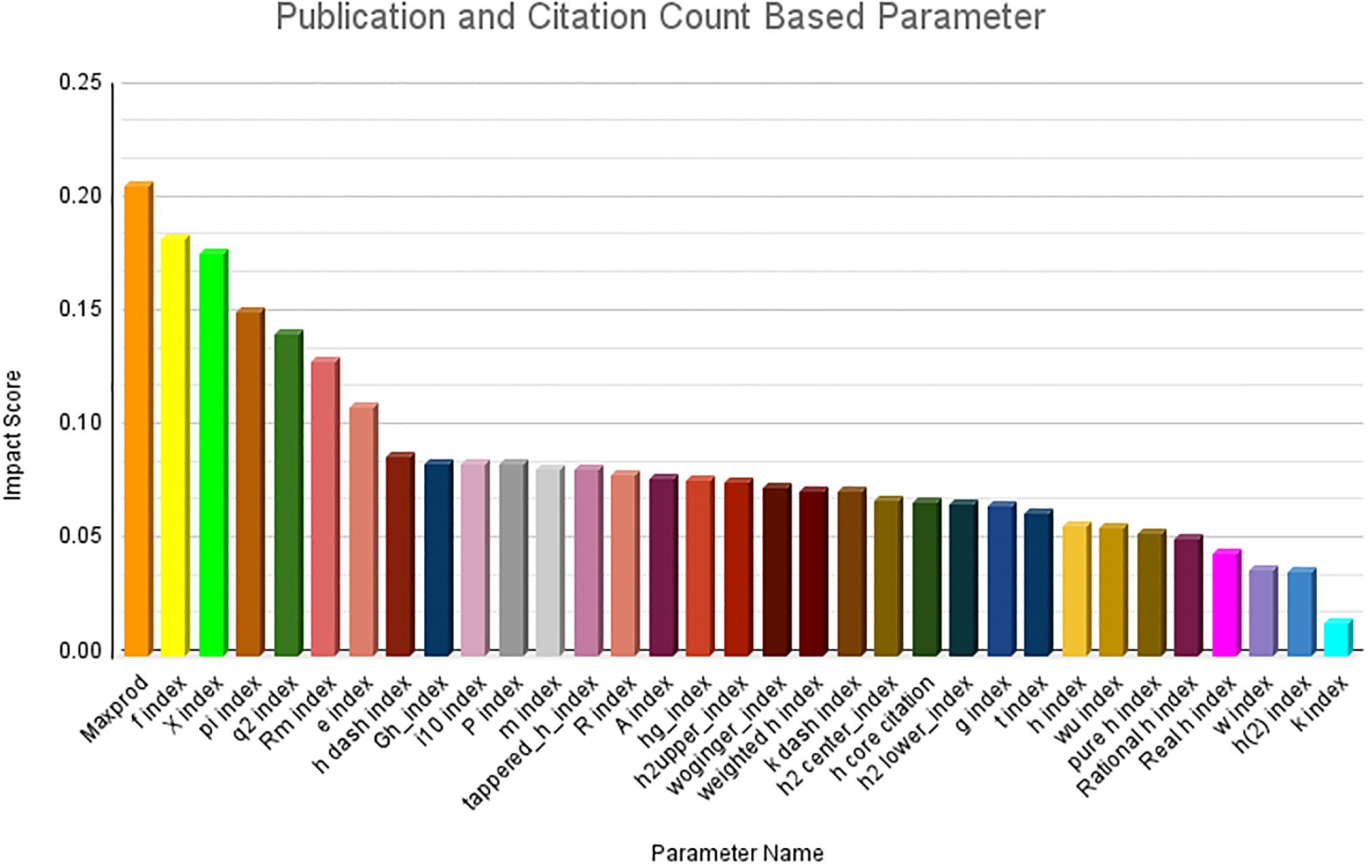
Publication and citation ranking.

**Fig 8:**
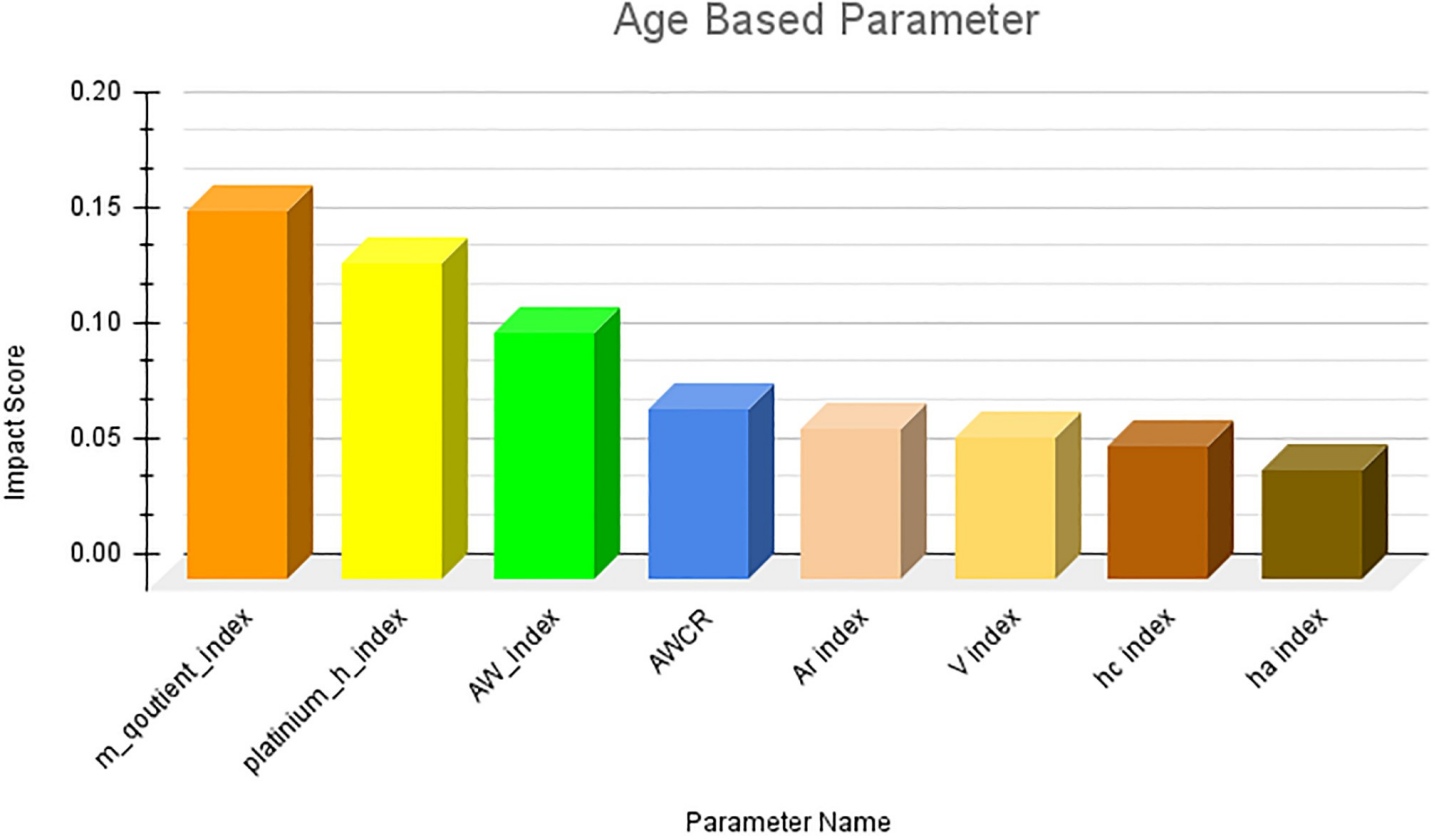
Age based parameter.

**Fig 9:**
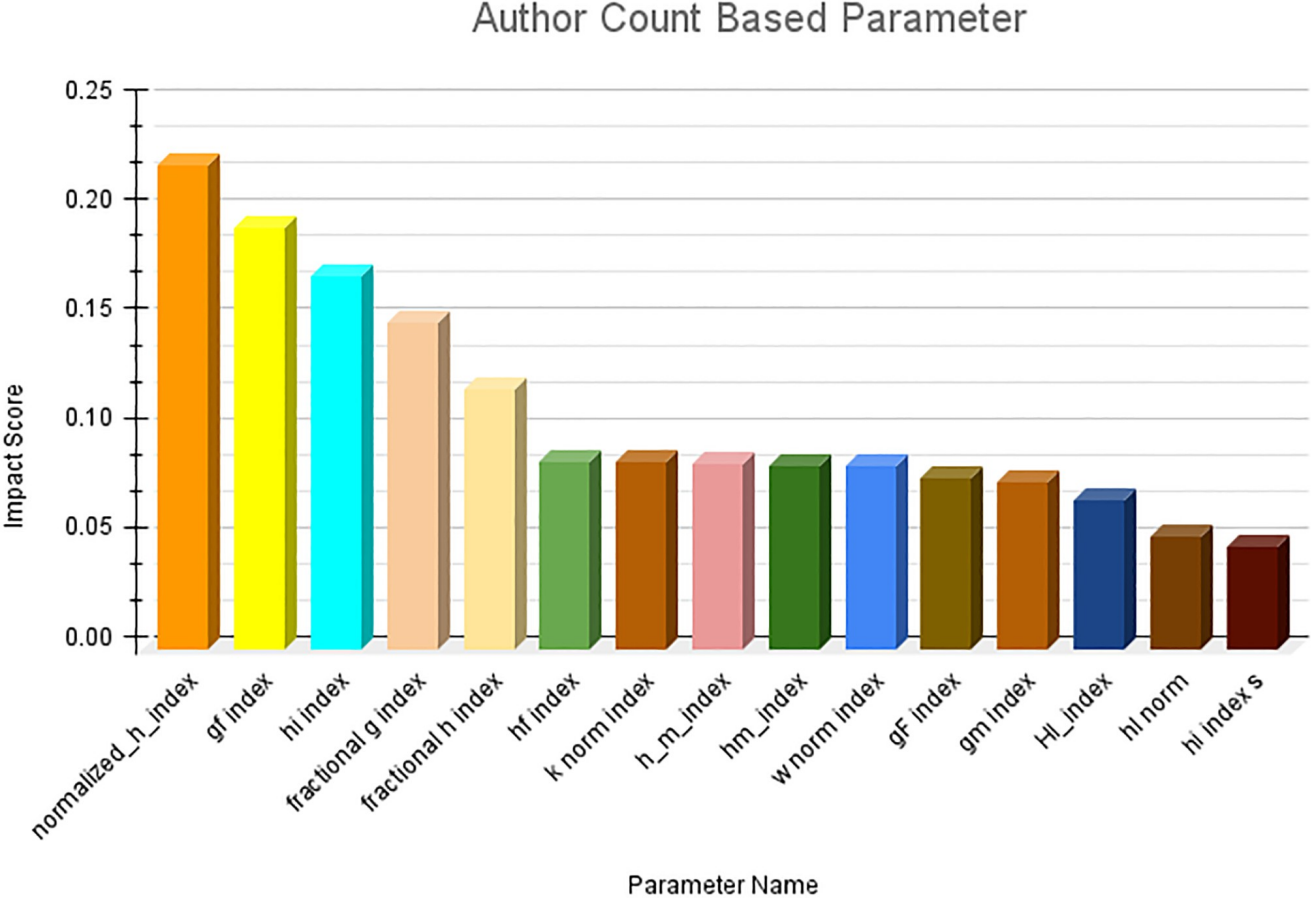
Author count based parameter ranking.

**Fig 10:**
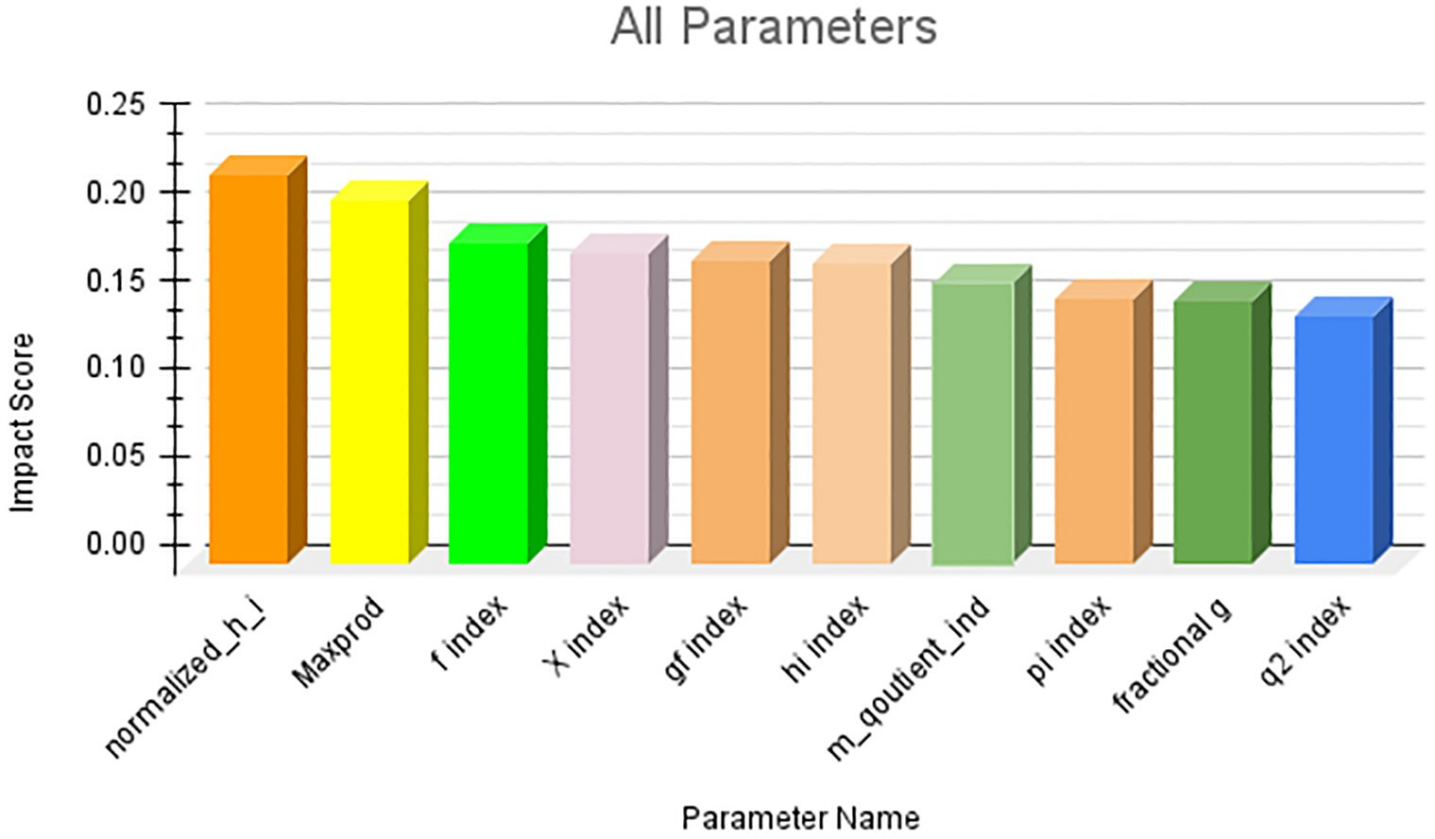
Top 10 highest impact score parameters.

The results obtained from the first research question are presented in this section. Owing to the large number of parameters, it is not feasible to present them in a single figure. Therefore, we first ranked the parameter categories wise and subsequently consolidated the top 10 ranked parameters across all categories. The category-wise results are shown in Figs 6–9. The results clearly indicate that among the primitive parameters, total citations had the highest impact score of nearly 0.13. In terms of publication- and citation-based parameters, the Maxprod parameter showed the highest impact score compared to others, at 0.20. Within the age-based category, the m-quotient demonstrated the highest impact score of 0.15. Lastly, in the author count-based category, the normalized h index achieves the highest score of 0.22. In Fig 10, we have combined all the category parameters and presented the top 10 parameters with the highest impact scores. Based on the figure, we have identified that the normalized h index outperforms all other parameters and is recognized as the most impactful parameter among the 64 parameters. The Maxprod parameter takes the second position among the top parameters, while the f index ranks third.

### Statistical evaluation

**Fig 11:**
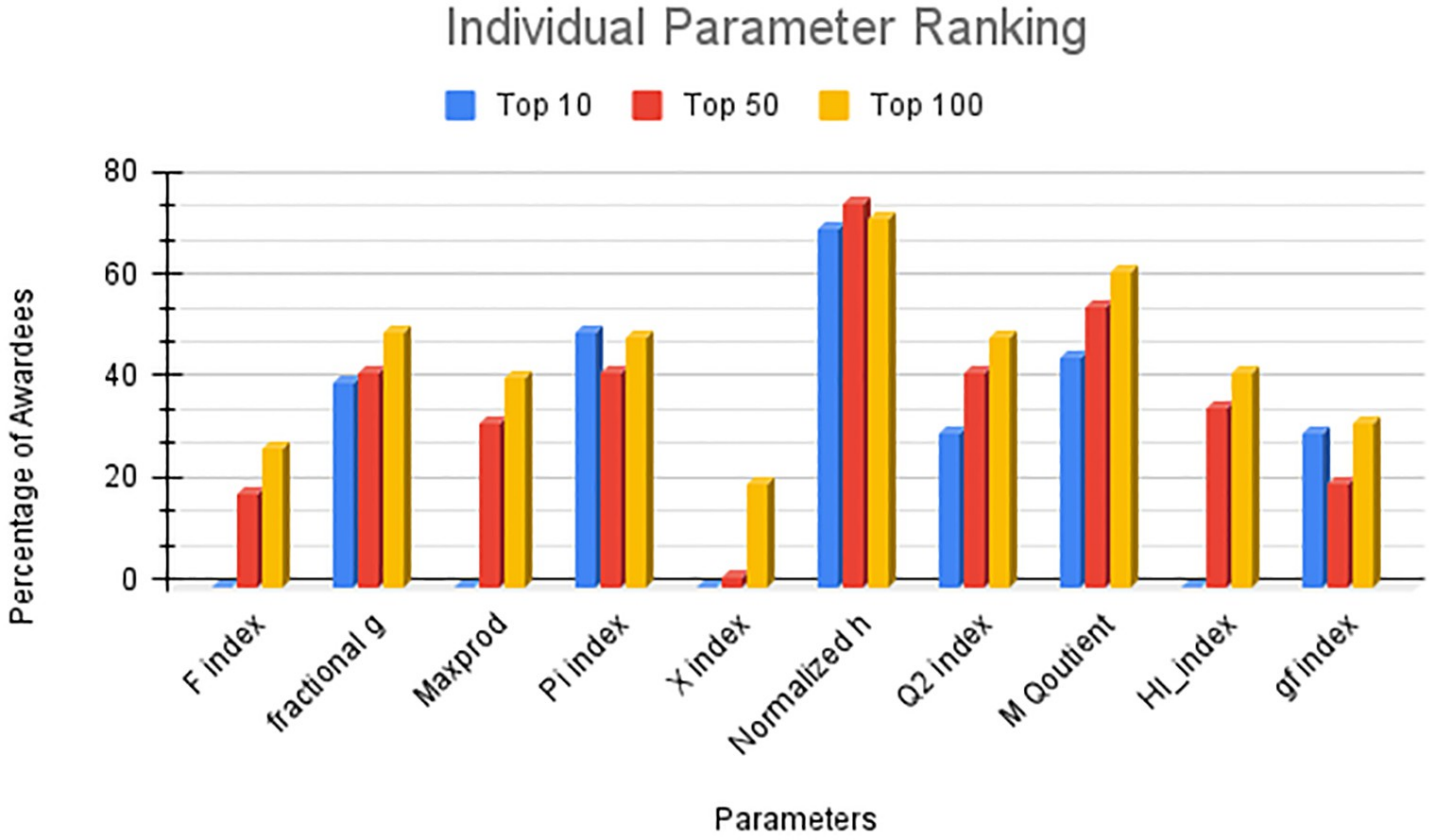
Individual parameter ranking.

Before conducting a statistical evaluation, it is important to assess the top ten ranked parameters individually. This initial evaluation allowed us to gain insights into the significance of each parameter. Subsequently, various statistical methods can be employed to combine these parameters and perform a comprehensive analysis of the results. To understand the impact of these parameters, we can examine the occurrences of awardees within the top 10, 50, and 100 records associated with these parameters. To facilitate this analysis, we first sorted the index data and then determined the number of awardees brought forth by these indices in the top 10, 50, and 100 records, respectively. Fig 11 shows the individual parameter ranking. As shown in the figure, the normalized h index outperformed all other parameters by bringing 70%, 72%, and 75% award recipients in the range of 10, 50, and 100 records, respectively. The m quotient bought 40%, 48%, and 59% of the awardees in the list of 10, 50, and 100 records, respectively. The X index performed worst by bringing 2%, 10%, and 20% awardees in the ranges of 10, 50, and 100 records, respectively.

After analyzing the individual performance of the top 10 highest-ranked parameters, the next crucial step involved combining these parameters with their various possible combinations across eight different statistical models. This process aims to explore and evaluate the collective impact of these parameters on the overall performance of awardees in top records. By employing this comprehensive approach, we can gain valuable insights into the interplay between different parameters and statistical models, thereby enhancing our understanding of their combined effects. First, we begin by generating all possible combinations of the top 10 indices and pairing them together. Once we have these pairs, our next step is to calculate the values of eight different statistical methods for each pair of parameters. After obtaining the values for each statistical method, we sorted the list of the values generated by each method. After sorting, to further investigate the performance of these statistical methods, we conducted an analysis of the top 10, 50, and 100 records. This analysis involved examining the number of awardees returned by each statistical method within these subsets of data. By observing the outcomes, we can evaluate the effectiveness of statistical methods for identifying awardees. Tables 4–6 present the top 10, 50, and 100 records of the analysis results, respectively. In these tables following abbreviations are used First Parameter of Pair (FP), Second Parameter of pair (SP), Arithmetic Mean (AM), Harmonic Mean (HM), Contra-Harmonic Mean (CHM), Geometric Mean (GM), Logarithmic mean (LOM), Lehmer mean (LM),Root Mean Square (RMS), Trigonometric mean (TM), F index (F), Fractional g index (FG), Maxprod (M), X index (X), Normalized h index (NH), Q2 index (Q), M Qoutient (MQ), Hi index (HI), gf index (GF) and *π* index (P).

**Table 4:** Top 10 records analysis results.

Parameters Pair	FP	SP	AM	HM	CHM	GM	LOM	RMS	TM
F & M	0	0	0	0	0	0	0	10	60
F & P	0	50	0	20	0	0	10	0	50
F & X	0	0	30	30	20	30	30	0	30
F & NH	0	70	20	0	20	0	0	20	30
F & MQ	0	45	20	100	20	100	70	20	80
F & HI	0	0	20	40	20	20	10	20	70
F & GF	0	30	10	0	20	0	10	20	60
F & Q	0	30	20	30	0	20	10	0	50
F & FG	0	40	10	0	0	10	0	0	60
M & P	0	50	0	0	0	0	10	0	70
M & X	0	0	40	10	60	20	20	70	80
M & NH	0	70	0	0	0	0	0	0	60
M & MQ	0	45	0	100	0	90	70	0	100
M & HI	0	0	0	40	0	10	20	0	60
M & GF	0	30	0	0	0	0	10	10	60
M & Q	0	30	10	0	20	10	10	70	60
M & FG	0	40	0	0	0	0	0	100	30
P & X	50	0	0	40	0	30	30	0	30
P & NH	50	70	0	0	0	0	10	0	60
P & MQ	50	45	0	100	0	90	60	0	90
P & HI	50	0	0	40	0	10	20	0	20
P & GF	50	30	0	0	0	0	10	0	60
P & Q	50	30	0	30	0	10	10	0	50
P & FG	50	40	0	30	0	10	10	0	50
X & NH	0	70	50	0	50	20	20	50	60
X & MQ	0	45	50	100	50	100	80	50	100
X & HI	0	0	50	40	50	30	40	50	60
X & GF	0	30	40	0	60	20	20	60	60
X & Q	0	30	40	30	50	30	20	70	50
X & FG	0	40	50	40	50	40	20	60	60
NH & MQ	70	45	0	100	0	100	70	0	100
NH & HI	70	0	0	20	0	0	10	10	50
NH & GF	70	30	0	0	10	0	10	40	60
NH & Q	70	30	30	0	30	10	10	30	40
NH & FG	70	40	30	0	40	0	0	40	60
MQ & HI	45	0	50	100	30	100	80	30	100
MQ & GF	45	30	0	100	0	100	80	0	100
MQ & Q	45	30	30	100	30	90	70	30	100
MQ & FG	45	40	30	100	30	100	70	30	90
HI & GF	0	30	0	30	0	10	20	0	70
HI & Q	0	30	30	40	30	10	10	30	60
HI & FG	0	40	30	40	30	30	20	30	50
GF& Q	30	30	20	0	30	10	10	30	60
GF& FG	30	40	20	0	40	0	0	50	80
Q & FG	30	40	20	10	20	0	0	90	40

**Table 5:** Top 50 records analysis results.

Parameters Pair	FP	SP	AM	HM	CHM	GM	LOM	RMS	TM
F & M	18	32	6	10	12	10	10	16	46
F & P	18	42	32	4	32	20	20	32	48
F & X	18	2	16	36	18	30	22	18	40
F & NH	18	75	4	2	4	2	4	4	50
F & MQ	18	55	4	85	4	88	64	4	76
F & HI	18	35	4	48	4	18	18	4	70
F & GF	18	20	4	24	4	4	6	4	54
F & Q	18	42	10	16	10	12	10	14	52
F & FG	18	42	6	28	8	18	10	12	56
M & P	32	42	32	16	32	24	20	32	44
M & X	32	2	34	20	42	24	24	54	54
M & NH	32	75	12	4	18	8	8	18	50
M & MQ	32	55	16	86	16	82	68	16	82
M & HI	32	35	16	48	18	30	28	18	60
M & GF	32	20	18	20	16	20	12	16	70
M & Q	32	42	12	10	18	8	8	42	54
M & FG	32	42	24	24	30	24	16	94	46
P & X	42	2	32	44	32	36	34	32	40
P & NH	42	75	32	2	32	16	12	32	56
P & MQ	42	55	32	81	32	74	58	32	82
P & HI	42	35	32	48	32	36	30	32	52
P & GF	42	20	32	28	32	24	26	32	56
P & Q	42	42	32	18	32	22	20	32	46
P & FG	42	42	32	42	32	38	22	32	46
X & NH	2	75	42	4	42	16	12	42	64
X & MQ	2	55	44	85	42	84	60	42	84
X & HI	2	35	44	48	42	40	34	42	74
X & GF	2	20	44	28	42	34	28	44	60
X & Q	2	42	38	22	40	28	18	66	66
X & FG	2	42	44	36	46	38	22	48	56
NH & MQ	75	55	2	80	2	80	64	2	90
NH & HI	75	35	6	36	2	16	18	2	72
NH & GF	75	20	18	4	30	4	6	44	54
NH & Q	75	42	18	4	20	8	8	20	54
NH & FG	75	42	38	4	46	16	12	46	58
MQ & HI	55	35	56	80	46	84	66	40	88
MQ & GF	55	20	28	83	24	84	72	24	84
MQ & Q	55	42	20	79	20	78	64	20	80
MQ & FG	55	42	42	84	42	86	62	42	74
HI & GF	35	20	26	48	26	36	28	26	72
HI & Q	35	42	20	48	20	24	22	20	64
HI & FG	35	42	42	48	42	42	36	42	70
GF & Q	20	42	18	22	20	14	12	22	62
GF & FG	20	42	32	26	44	28	22	46	68
Q & FG	42	42	22	16	24	10	14	52	66

**Table 6:** Top 100 records analysis results.

Parameters Pair	FP	SP	AM	HM	CHM	GM	LOM	RMS	TM
F & M	27	41	4	24	10	13	13	16	50
F & P	27	49	41	5	41	29	20	41	47
F & X	27	20	18	33	13	26	22	15	37
F & NH	27	72	3	20	4	11	8	4	55
F & MQ	27	62	2	74	2	76	54	2	65
F & HI	27	42	2	58	2	36	30	2	66
F & GF	27	32	4	34	3	14	15	4	52
F & Q	27	49	9	16	10	14	9	14	53
F & FG	27	50	4	37	10	19	15	12	49
M & P	41	49	41	27	41	37	32	41	44
M & X	41	20	38	25	41	34	27	53	53
M & NH	41	72	26	20	28	21	21	28	49
M & MQ	41	62	27	77	27	71	59	27	59
M & HI	41	42	27	58	27	44	34	27	60
M & GF	41	32	27	34	27	30	25	27	59
M & Q	41	49	24	21	24	22	22	37	53
M & FG	41	50	37	33	38	36	25	88	48
P & X	49	20	41	42	41	42	33	41	49
P & NH	49	72	41	20	41	33	25	41	49
P & MQ	49	62	41	93	41	74	56	41	64
P & HI	49	42	41	58	41	45	38	41	57
P & GF	49	32	41	34	41	40	30	41	55
P & Q	49	49	41	23	41	32	25	41	48
P & FG	49	50	41	45	41	42	27	41	46
X & NH	20	72	42	19	43	26	20	43	53
X & MQ	20	62	42	75	42	75	55	42	69
X & HI	20	42	42	57	42	46	34	42	64
X & GF	20	32	40	32	43	37	28	42	54
X & Q	20	49	35	29	38	32	26	62	56
X & FG	20	50	41	41	42	42	28	46	55
NH & MQ	72	62	22	78	17	73	49	16	77
NH & HI	72	42	23	53	16	42	31	13	62
NH & GF	72	32	28	23	34	24	17	39	62
NH & Q	72	49	22	20	24	19	19	24	54
NH & FG	72	50	42	21	47	30	19	47	54
MQ & HI	62	42	66	96	55	77	55	50	78
MQ & GF	62	32	36	75	33	74	62	33	68
MQ & Q	62	49	23	77	22	79	61	22	70
MQ & FG	62	50	45	76	45	84	58	45	69
HI & GF	42	32	35	57	34	48	38	33	61
HI & Q	42	49	23	58	22	41	32	22	68
HI & FG	42	50	45	58	45	49	38	45	64
GF & Q	32	49	23	31	22	28	21	25	62
GF & FG	32	50	44	34	45	37	28	46	62
Q & FG	30	50	33	31	35	13	23	40	60

The Tables 4–6 present the percentage scores of awardees for the individual parameters and their combinations using different statistical models for the top 10, 50, and 100 records. In the following paragraphs, we analyze different statistical models in relation to various parameter combinations.

### Arithmetic mean (AM)

- In the top 10 records, the Arithmetic Mean (AM) demonstrated moderate percentage scores ranging from 0% to 50% for all parameter combinations. The AM scores tended to be notably lower than the individual parameter scores in most parameter combinations. For the given dataset, the highest AM score recorded was 50%, which was achieved by using several parameter combinations. Notable examples include the combination of ‘HI index’ and ‘Normalized h index’ as well as the combination of ‘M Quotient’ and ‘X index.’
- Further examining the top 50 records, the behavior of the Arithmetic Mean (AM) exhibited trends similar to those observed in the previous analysis. Among these records, the highest percentage score achieved using the AM was 44%. This notable score is obtained by the parameter combination o of m Quotient’ and ‘X index, while the lowest percentage score recorded in this subset is 2%, achieved by M Quotient’ and ‘Normalized h index.
- Extending the analysis to the top 100 records, the trend observed with the Arithmetic Mean (AM) remained consistent. However, there was a slight increase in the percentage scores of up to 66 percent achieved by the M Quotient and HI index. Conversely, the lowest accuracy score recorded for this subset is 2%. This score is achieved by combining the F index’ and ‘HI index parameters.

### Harmonic mean (HM)

- Examining the top 10 records, it becomes evident that the Harmonic Mean (HM) performs better than the Arithmetic Mean (AM) in terms of the award percentage score. The HM yields better results than the AM, with some instances where the HM score drops to zero when combining certain indices, in contrast to their individual scores. Interestingly, when combining t the m Quotient’ index with any other index using HM, the resulting accuracy score was consistently 100 percent. Furthermore, most of the indices returned a 0 percent result, except for the M Quotient combination.
- Expanding the analysis to the top 50 records, the Harmonic Mean (HM) continued to exhibit a slightly lower performance compared to the previous subset. However, the behavior of t the m Quotient’ index when combined with other indices remains consistent and achieves higher percentage scores, reaching up to 86% percentage score by ‘M Quotient’ and’ Maxprod.’ The lowest percentage score recorded in this subset was 2%, achieved by the F index and the Normalized h index.
- Extending the analysis to the top 100 records, the trend observed with t the m Quotient parameter in conjunction with the Harmonic Mean (HM) remains consistent. However, there was a slight decrease in the percentage scores, which was likely due to an increase in the number of records. Within this extended subset, the highest accuracy score achieved using the HM was 77%. This notable score was obtained by combining t the m Quotient’ parameter with the ‘Q2 index. Conversely, the lowest accuracy score recorded for this subset is 5%. This score is achieved by combining the F index’ and’ p index parameters.

### Contra harmonic mean (CHM)

- Among the top 10 records, the Contra harmonic mean (CHM) exhibited a similar behavior to the Arithmetic Mean (AM), with percentage scores ranging between 0% and 60% for all parameter combinations. In this subset, the highest percentage score achieved using the CHM was 60%. This notable score is attained by the combination of t the m index’ and ‘X index.’ This suggests that when evaluated using the CHM, this specific combination yields a relatively higher level of accuracy than the others. Conversely, the lowest percentage score recorded in this subset was 0%, which was obtained using many combinations.
- Expanding the analysis to the top 50 records, the Contra Harmonic Mean (CHM) continued to demonstrate a slightly lower performance compared to the previous subset. The percentage scores ranged from 0% to 46% for the various parameter combinations. Among these records, the highest percentage score achieved using the CHM was 46%. This notable score is obtained by multiple combinations, one of which is the combination of the M Quotient’ and ‘HI index. The lowest percentage score recorded for this subset was 2%. This score is achieved by combining the ‘HI index’ and the Normalized h index.
- Extending the analysis to the top 100 records, the observed trend regarding percentage scores remained consistent with the previous dataset. The scores ranged between 2% and 55%, reflecting the performance of the Contra Harmonic Mean (CHM) for different parameter combinations. Within this extended subset, the highest accuracy score achieved using the CHM was 55%. This notable score was attained by combining t the m Quotient’ parameter with the ‘HI index. Conversely, the lowest accuracy score recorded in this subset is 2%. This score is achieved by combining the ‘F index’ and ‘HI index.

### Geometric Mean (GM)

- Examining the top 10 records, it becomes evident that the Geometric Mean (GM) exhibits a similar performance to the Harmonic Mean when it comes to combinations involving t the m Quotient’ index. Remarkably, when combining t the m Quotient’ index with any other index using GM, the resulting accuracy score consistently falls within the range of 90% to 100%. Conversely, most of the other indices, when combined with any other index and assessed using GM, tended to return accuracy scores of 0%.
- Expanding the analysis to the top 50 records, the GM continued to exhibit a slightly lower performance compared to the previous subset. However, the behavior of the ‘M Quotient’ index when combined with other indices remains consistent and achieves higher percentage scores, reaching up to 88% percentage score by ‘M Quotient’ and ‘F index.’ The lowest percentage score recorded in this subset is 2 percent achieved by F index and ‘Normalized H -index.
- Extending the analysis to the top 100 records, the trend observed with t the m Quotient’ parameter in conjunction with GM remains consistent. However, there is a slight decrease in the percentage scores, likely due to the increase in the number of records. Within this extended subset, the highest accuracy score achieved using the HM was 79%. This notable score is obtained by combining the ‘M Quotient’ parameter with the ‘Q2 index’. Conversely, the lowest accuracy score recorded for this subset is 11%. This score is achieved by combining the parameters ‘F index’ and ‘Normalized h index.

### Logarithmic Mean (LOM)

- Examining the top 10 records, it is evident that the behavior of t the m Quotient’ index is consistent with the Logarithmic Mean (LM). Combinations involving t the m Quotient index generally result in a percentage of awardees ranging from 0% to 80%. The highest was achieved by the M Quotient and the HI index. Conversely, when most other indices were combined with any other index and assessed using the LOM, they tended to return percentage scores of 0%.
- Expanding the analysis to the top 50 records, the result of the M Quotient combination result becomes dominant over other combinations. The highest percentage was obtained by the M Quotient’ and the gf index, which was 70. The lowest percentage score recorded in this subset was 4%, achieved by the F index and ‘Normalized h index.
- Extending the analysis to the top 100 records, the trend observed with t the m Quotient’ parameter in conjunction with LOM remains consistent. However, there is a slight decrease in the percentage scores, likely due to the increase in the number of records. Within this extended subset, the highest accuracy score achieved using the HM was 62%. This notable score was obtained by combining t the m Quotient’ parameter with the ‘gf index. Conversely, the lowest accuracy score recorded for this subset is 8%. This score is achieved by combining the parameters ‘F index’ and ‘Normalized h index.

### Root Mean Square(RMS)

- Examining the top 10 records, it is evident that the combination involving the ‘FG index’ demonstrates dominance over other combinations by achieving a perfect score of 100%. Additionally, when the ‘FG index’ is combined with the ‘Q2 index, it retrieves a percentage score of 90%. Conversely, some of the other indices are combined with other indices and assessed using the Root Mean Square (RMS); they tend to return percentage scores of 0%.
- Expanding the analysis to the top 50 records, the result of the FG index with Maxprod became dominant and attained a percentage of 84. The lowest percentage score recorded in this subset is 4 percent achieved by F index and ‘Normalized h index.
- Extending the analysis to the top 100 records, the trend observed with the FG index parameter, in conjunction with the RMS, remains consistent. However, there is a slight decrease in the percentage scores, likely due to the increase in the number of records. Within this extended subset, the highest accuracy score achieved using the RMS was 78%. This notable score was obtained by combining the ‘Maxprod’ parameter with the ‘gf index.’ Conversely, the lowest accuracy score recorded for this subset is 4%. This score is achieved by combining the parameter ‘F index’ and the M Quotient.

### Trigonometric Mean(TM)

- Examining the top ten records, the use of the Trigonometric Mean (TM) yielded astonishing results. Unlike other statistical methods, no single combination returns a 0 percent accuracy score. This suggests that the TM mean performs consistently well across the different parameter combinations. The lowest accuracy score recorded in this subset was 30 percent, achieved by combining the ‘F index’ and ‘X index.’ On the other hand, the highest accuracy score of 100 percent is attained by combining the ‘M Quotient’ and ‘Maxprod’ indices.
- Expanding the analysis to the top 50 records, it is evident that the Trigonometric Mean (TM) continues to exhibit consistent results across different combinations. Regardless of the parameter combination, the TM mean maintained stable performance. In this subset, the highest accuracy score achieved by using the TM mean was 90 percent. This notable score was obtained by combining t the m Quotient’ index with the ‘HI index. In contrast, the lowest accuracy score recorded in this subset is 40 percent, achieved by combining the ‘Pi index’ and ‘X index.’
- Extending the analysis to the top 100 records, the trend observed in the Trigonometric Mean (TM) remains consistent. TM continues to demonstrate stable performance across various combinations within this extended subset. In this subset, the highest accuracy score achieved using the TM was 78 percent. This notable score was obtained by combining t the m Quotient’ and ‘HI indices. The lowest percentage score recorded for this subset was 37 percent. This score is achieved by combining the F and X indices.

Based on a comprehensive analysis of the statistical methods applied to the parameter combinations, it becomes apparent that the Trigonometric Mean (TM) outperforms the other six statistical models. TM consistently produces exceptional results when evaluating the percentage of different parameter combinations. Throughout the analysis, TM consistently demonstrated its effectiveness in capturing the percentage score across various combinations. Its performance remains stable and provides notable accuracy scores, even when compared with other statistical models. The exceptional results obtained with TM suggest that it is a robust statistical method for evaluating accuracy within a given dataset. It exhibits a unique ability to capture the underlying relationships and patterns between parameters, resulting in a higher percentage score. Based on the analysis of the parameters, namely the M Quotient and FG index, it is evident that these two indices exhibit prominent performance across different combinations and statistical models. When combined with other parameters using various statistical methods, the M Quotient and FG index consistently yielded excellent results in terms of the percentage score for returning awardees. The exceptional performance of the M Quotient and FG indices suggests that these indices possess significant predictive power or are strongly correlated with the desired outcome. Their combination with other parameters led to consistently high accuracy scores across the different statistical models. Moreover, in some cases, the normalized h index performs well.

## Conclusion

This study involved a thorough analysis of author assessment parameters, encompassing sixty-three different parameters categorized into four distinct categories. The dataset employed in this study consisted of 525 non-awardee authors and 525 awardee authors from prestigious scientific societies in the mathematics domain. Given the significant number of parameters, we propose a Modified Recursive Elimination technique to rank these sixty-three parameters. For classification purposes, we used a multilayer perceptron classifier algorithm. This algorithm generates importance scores for each parameter, which are then used to rank them accordingly. The ranking results indicated that the normalized h index surpassed all other parameters in terms of performance. This signifies that the importance score of the normalized h index outweighs that of all the other parameters, highlighting the effectiveness of this particular index. In addition, we selected the top 10 parameters with the highest rankings and conducted statistical analysis. For this analysis, we used seven statistical methods. These methods were employed to combine the top ten parameters for all possible combinations. Subsequently, we sorted the resulting combination lists based on the values obtained through statistical methods. From these lists, we performed analyses on the top 10, 50, and 100 records. In these analyses, we examined the occurrence of awardees within each list of the top records. The findings of the analysis revealed that the Trigonometric Mean (TM) outperformed the other six statistical models. Furthermore, parameter analysis demonstrated that the M Quotient and FG index consistently produced significant results across various combinations and statistical models. When combined with other parameters using different statistical methods, the M Quotient and FG index consistently yielded excellent percentage scores for predicting awareness. Additionally, the normalized h index performed well in certain cases. The limitation of this study is that the presented result is only applicable to the mathematics domain; whenever the field changes, the result may change.

## Future work

In future endeavors, we will expand the scope of our research in multiple dimensions. Firstly, we are incorporating additional new published indices into our list of metrics, such as the Kaptay K index, the H Alpha-index, the Psi index, and numerous others. Secondly, we will incorporate multiple domain datasets such as Civil Engineering, Neuroscience, and Computer Science and so on.

## Supporting information

S1 Appendix Indices calculation formulas [4, 7, 8, 19, 21, 24, 35–70]. (PDF)
