# Supplementary material for: MRET: Modified Recursive Elimination Technique for ranking author assessment parameters
Source: PLoS One. 2024 Jun 13;19(6):e0303105. doi: 10.1371/journal.pone.0303105 (PMC11175400; doi:10.1371/journal.pone.0303105)
Supplement: S1 Appendix — (PDF) [file pone.0303105.s001.pdf]

Appendix A

Table 7. Indices calculation Formulas

**Name of Index**	**Calculation**
Total Publication [35]	Total No of Publication of a researchers
Total Citation [36]	Total No of Citation of a researchers
Total Years [24]	Number of years since the academic's first publication
Cites per Year [24]	Total citations/years since first paper
Cites per Paper [24]	Total citations/total papers
Author per Paper [24]	Add up the total number of authors involved in the publications for the author in question and divide this by the number of papers.
Cites per author [24]	Divide citations for each publication by the number of authors and sum the resulting citations; this is the single-authored equivalent number of citations for the author in question
Papers per author [24]	Divide each publication by the number of authors and sum the fractional author counts; this is the single-authored equivalent number of papers for the author in question.
h index [8]	h = max (numbers of articles with >= h citations)
G-index [37]	A set of papers has a g-index g if g is the highest rank such that the top g papers have, together, at least g2 citations.
Hg-index [21]	$hg - index = \sqrt{h * g}$<br>Where h represent h index and g represent g index.
A-index [38]	$A - index = \frac{1}{h}\sum_{p=1}^{h} cit_p$<br>Where A is the A-index of the scholar, h represents the h index and cit_p is the citation count of the pth article.
R-index [19]	$R - index = \sqrt{\sum_{p=1}^{h} Cit_p}$<br>Where h represents the h index and cit_p is the citation count of the pth article.
P-index [35]	$P - index = (\frac{C^2}{p})^{\frac{1}{3}}$<br>The p-index strikes the best balance between total number of citations (C) and the mean citation rate (C/P)
Q^2-index [39]	$Q^2 = \sqrt{h * m}$<br>Where h represent h index and m represent m index
K-index [40]	$K - index = \frac{\frac{C}{P}}{\frac{C(h-tail)}{C(h-core)}}$<br>Where C represent total citation, p represents of pth article, h-tail represent h-tail article citation and h-core represent h-core citation.
E-index [41]	$e - index = \sum_{p=1}^{h} Cit_p - h^2$

	Where Cit_p represent citation of pth article and h represent h index
f-index [42]	$f-index = \binom{max}{f} \frac{1}{\frac{1}{f}\sum_{p=1}^{f}\frac{1}{Cit_p}} \geq f$ Where cit_p is the citation count of pth article. The f-index never goes beyond the total number of publications.
T-index [43]	$T-index = \binom{max}{t} exp\left[\frac{1}{t}\sum_{k=1}^{t} ln(Cit_k)\right] \geq t$ Where cit_k is the citation count of kth article
Tappered h index [44]	$H_{T(j)} = \frac{n_j}{2_j-1}, \quad n_j \leq j$ and $h_{T(j)} = \frac{j}{2_j-1} + \sum_{i=j+1}^{n_j}\frac{1}{2_j-1}, \quad n_j > j$
Wu index [7]	The w-index of an author is calculate, as if at least w of their articles have garnered 10w citations, while the remaining publications have received less than 10(w+1) citations each.
Weighted h index [4]	$R_w(k) = \frac{\sum_{p=1}^{k} Cit_p}{h}$ Where h is the h index and cit_p is the citation count of the pth article. Then, the weighted h index. Is defined as follows $h_w = \sqrt{\sum_{k=1}^{R} Cit_k}$ Where cit_k is the citation count of the kth article and R is the largest rank among all publications such that the kth weighted rank $< cit_k$.
h(2)-index [45]	The h(2)-index is the maximum whole number such that the h(2) most cited articles by a scholar have each received at least $(h(2))^2$ citations.
Woeginger index [46]	$w = \binom{max}{w}(Cit_p \geq w-p+1) \quad for\ all\ \ p \leq w$ where $cit_p$is the citation count of pth article and w is the maximum number of publications.
Rm-index [47]	$R_m = \sqrt{\sum_{k=1}^{h} \mathrm{Cit}_k^{\frac{1}{2}}}$ Where cit_k is the citation count of kth article.
m-index [48]	A m-index is calculated as the median number of citations received by the h-core articles.
X-index [49]	$x = \sqrt{\binom{max}{k} kCit_k}$ Where cit_k is the citation count of kth article.
h2 upper-index [49]	$h^2upper = \frac{\sum_{k=1}^{h}(Cit_k-h)}{\sum_{k=1}^{m} Cit_k} * 100 = \frac{e^2}{\sum_{k=1}^{m} Cit_k} * 100$ Where h is the h index, cit_k is the citation count of the kth article, $e^2$is the excess citation and m is the total number of articles.
h2 center-index [49]	$h^2center = \frac{h*h}{\sum_{k=1}^{m} Cit_k} * 100$ Where h is the h index and cit_k is the citation count of the kth article
h2 lower-index [49]	$h^2lower = \frac{\sum_{k=h+1}^{m}(Cit_k-h)}{\sum_{k=1}^{m} Cit_k} * 100$ Where h is the h index and cit_k is the citation count of the kth article
h` –index (h dash) [50]	$h' = Rh = \frac{eh}{t}$

	where R represents the head-tail ratio of e and t-index
Rational h index [51]	$h_{rat} = h + 1 - \frac{k}{2h+1}$<br><br>Where h is the h index and k is the number of citations required to reach h+1 h index value.
I10 index [52]	This is a simple and direct measure of indexing that involves counting the total number of papers published by a journal that have received at least 10 citations.
Normalized h index [53]	$normalized\ hindex = \frac{h}{pub_{count}}$<br><br>Where h represents h index and pubcount represent total publication.
Π *index* [54]	$\Pi\ index = 0.01C(P\Pi)$<br>The $\Pi - index$ is equal to the 100th of the number of citations, $C(P\pi)$ to the top square root $(P\pi)$ of the total papers(P).
Gh index [55]	$Gh^a = \sum_{p=1}^{m} sing(Cit(\text{pub}_b^a) - GH) \quad where\ \ sing(x) = 1, x \geq 0\ and\ \ 0, x \leq 0$<br>Where m is the total number of publications of scholar a and GH is the h index of the scholar. This index is also difficult to compute in comparison to the h index.
W index [38]	w-index is defined as w, which represents the number of their top articles that have at least 10w citations each. While w-index can be a useful measure for finding impact of scholar, it may penalize young scholars who have recently started working or those who have not yet published enough papers.
Maxprod [56]	The maximum value of i * c_i can be found by examining the publication rank of an author, where c_i represents the number of citations for the ith most frequently cited paper among all the citations.
H core citation [24]	The h-core citation index takes into account only those publications that have been cited at least h times and ignores those that have not achieved this threshold
K dash index [57]	$k^{'} = \frac{Cit_{all} - Pub_{Count}}{Cit_T - Cit_H}$<br>Where cit_{all} represent total citation, pub_{count} represent total publication, cit_t represent total citation of h tail article and cit_h represent total citation of h core article.
M-Quotient [15]	$M - Quotient = \frac{hindex}{y}$<br>Where y represent no of the year the first publication
Hc – index [53]	$hc\text{-}index = \alpha \cdot \frac{C(i)}{(Y(\text{now}) - Y(i) + 1)}$<br>Where $Y(\text{now})$ represents the current year, $Y(i)$ represents the publication year, and $C(i)$ represents the paper i citation count.<br>$hc\text{-}index = \frac{C(i)}{1}, \frac{C(i)}{2}, \frac{C(i)}{3}, \ldots, \frac{C(i)}{n}$
Aw-index [15]	$Aw\text{-}index = \sqrt{\sum_{j=1}^{h} \frac{Cit_j}{a_j}}$<br>Where Cit_j represents the citation count of the jth article and a_j represents the jth article, and m represents the total number of articles.

Ar index [19]	$Ar - index = \sqrt{\sum_{j=1}^{h} \frac{Cit_j}{a_j}}$ Where Cit_j citation of the article and a_j represent a^{th} article, h represent total h core article.
AWCR [58]	This parameter adjusts the citation count based on the length of time that has passed since each publication.
v-index [59]	$V = \frac{h}{P(y_{this} - y_o)}$ Where h is the h index, y_{this} is the current year and y_0 is the year of first publication.
Platinum h index [60]	$Platinium - h = \frac{H}{CL} * \frac{cit_{all}}{Pub_{count}}$ <br>Where H is the h index, CL is the career length, Cit_{all} is the total citation count and pub_{count} is the publication count.
Ha index [61]	The ha-index against a dataset is the largest number of papers in the dataset that have obtained at least ha citations per year on average.
HI Index [62]	$h_i = \frac{h}{Avg_a}$ where h represent h index and Avg_a represent average no of authors in article.
hf index [63]	$\frac{Yh_f}{\phi(Yh_f)} \geq h_f$ where Y(i) represent citation count and $\phi(i)$ represent average no of authors in article.
gf index [63]	$gf = \sum_{i=1}^{gf} \frac{Y_i}{\phi_i} \geq g^2 f$ where Y(i) represent citation count and $\phi(i)$ represent average no of authors in article.
gF index [63]	$gF = (\sum_{i=1}^{k} \frac{1}{\phi(i)})^2 \leq \sum_{i=1}^{k} y_i$ where Y(i) represent citation count and $\phi(i)$ represent average no of authors in article.
Normalized Hi index [64]	normalized hi index $= \frac{h}{pub_{count}}$ <br>Where h represents the h index and pub_{count} is the total number of articles. .
Hm index [65]	$r_{eff}(r) = \sum_{r'=1}^{r} \frac{1}{a(r')} thenc(r(h_m)) \geq h_m \geq c(r(h_m) + 1)$
k norm index [66]	$k - norm = h - norm + (1 - (h - \frac{norm^2}{\sum_{j=1}^{h-norm} citnorm_i})), \quad \forall h - norm > 1 \; and \; k - norm = 0, \; if \; h - norm = 0$
w norm index [67]	$w - norm = h - norm + (1 - (h - \frac{norm^2}{totalcit - norm})), \quad \forall h - norm > 0 \; and \; w - norm = \frac{totalcit - norm}{1 + totalcit - norm}, \; if \; h - norm = 0$
gm index [68]	$g_{m \leq} C_{eff}(g_m) \; where \; C_{eff}(r_{eff}) \; and \; S_{eff)}(r_{eff}) = \sum_{\acute{r}=1}^{r(r_{eff})} \frac{1}{a(\acute{r})} c(\acute{r})$
pure h index [63]	$h_p(A) = \frac{h}{\sqrt{E(author)}}$ Where h represent h index and E average no of author.
fractional h index [69]	$h_f = max(k \leq \frac{cit(k)}{author(k)})$ <br>Where Cit_k represent citation of article and author(K) represent no of author in a specific article.

fractional g-index [70]	$g_f = max(\sum_{k=1}^{p} \frac{cit_k}{Author(k)} \geq p^2)$
hi norm index [69]	The hl-norm is a modified version of the h index that normalizes citations based on the number of authors per paper.
K index [60]	$Kindex = \frac{c/p}{c(h-tail)/c(h-core)}$<br>Where C represent total citation, P represent total publication, C(h-tail) represent total citation of h tail article and C(h-core) represent total citation of h core article.
Real h index [70]	$h_r = \frac{(h+1)cit_h - h.cit_h + 1}{1 - cit_{h+1} + cit_h}$<br>Where h is the h index and cit_h is the citation count of the hth article.
